# The Role of Natural Compounds in Bladder Urothelial Carcinoma Treatment

**DOI:** 10.3390/ijms27020596

**Published:** 2026-01-07

**Authors:** Hangfei Jiang, Yueyin Chen, Xi Zeng, Rui Yang, Feng Zhang, Huanling Zhang, Erxiang Zhang, Xuzhang Wu, Deye Yan, Chunping Yu

**Affiliations:** 1 Department of Urology, The First Affiliated Hospital, Guangzhou Medical University, Guangzhou 510230, China; 2022111451@stu.gzhmu.edu.cn (H.J.); zhangfeng01@stu.gzhmu.edu.cn (F.Z.); 2 Guangdong Provincial Key Laboratory of Urological Diseases, Guangzhou Medical University, Guangzhou 510230, China; 3 Guangdong Engineering Research Center of Urinary Minimally Invasive Surgery Robot and Intelligent Equipment, Guangzhou Medical University, Guangzhou 510230, China; 4 Guangzhou Institute of Urology, Guangzhou Medical University, Guangzhou 510230, China; 5 The Third Clinical College, Guangzhou Medical University, Guangzhou 510230, China; 18219100443@163.com (Y.C.); zxuwzx@outlook.com (X.Z.); 2022111433@stu.gzhmu.edu.cn (R.Y.); 2023111317@stu.gzhmu.edu.cn (H.Z.); 2022111429@stu.gzhmu.edu.cn (E.Z.); 2022111434@stu.gzhmu.edu.cn (X.W.); 2022111415@stu.gzhmu.edu.cn (D.Y.)

**Keywords:** bladder urothelial carcinoma, natural products, apoptosis, cell cycle, invasion, drug resistance

## Abstract

Bladder urothelial carcinoma (BUC) ranks among the most common malignant tumors of the urinary system, with alarmingly high incidence and mortality rates. Current clinical treatments face challenges such as strong chemotherapy resistance and limited response rates to immunotherapy, creating an urgent need for novel alternative therapies. Natural products, characterized by multi-targeted antitumor activity, low toxicity, and broad availability, have emerged as highly promising adjunctive or alternative strategies in cancer treatment. Extensive research has elucidated the antitumor activities of natural products, including inhibition of cancer cell proliferation, induction of apoptosis, and modulation of the immune microenvironment. What’s more, their bioactive components, such as terpenoids and polyphenols, can synergistically enhance therapeutic efficacy while reducing toxicity risks associated with traditional therapies. This review will examine the roles of terpenoids, phenolics, alkaloids, and other natural products in BUC treatment, to provide directions for future research.

## 1. Introduction

According to global cancer statistics, BUC is the tenth most common cancer in the world, with approximately 573,000 new cases and 213,000 deaths each year [1]. The incidence of BUC has obvious gender differences, with male patients accounting for about 75%, and the incidence rate increases with age, with the highest incidence rate in people over 70 years old. BUC not only imposes a heavy medical burden on patients and their families but also leads to indirect economic burdens due to loss of productivity and reduced quality of life, further exacerbating its social impact.

At present, the main treatments for BUC are surgery, chemotherapy, and immunotherapy. Radical cystectomy is the standard treatment for muscle-invasive bladder cancer (MIBC), whereas transurethral resection of bladder tumor (TURBT) is commonly used for non-muscle-invasive bladder cancer (NMIBC). Radical cystectomy is the standard treatment for muscle-invasive bladder cancer (MIBC), whereas TURBT is commonly used for NMIBC [2]. Although there has been progress in surgical techniques, the recurrence rate of BUC is still high, especially for NMIBC patients, with a recurrence rate of up to 70% within five years [3]. To lower the chance of it coming back or getting worse after surgery, doctors might use other treatments after the operation. These include putting Bacillus Calmette-Guérin (BCG) or chemotherapy drugs inside the bladder through a tube. But they are not very effective and can cause serious side effects [4].

For advanced or metastatic BUC, cisplatin-based systemic chemotherapy is the mainstay of treatment. But the appearance of drug resistance is the primary cause of treatment failure and poor prognosis. In recent years, the development of immune checkpoint inhibitors has provided new hope for patients with advanced BUC. However, only some patients respond to immunotherapy, and the mechanism of resistance remains unclear [5]. Also, the high costs of these new treatments have prevented them from being used by many people, which makes it harder for everyone to get better when they have cancer.

Although there have been great advancements in cancer treatment in modern medicine, the limitations of traditional chemotherapy and radiotherapy, such as toxic side effects, drug resistance, and limited therapeutic efficacy, have led researchers to constantly seek out new therapeutic approaches. Natural products have become an important source for developing anticancer drugs because they have many different structures and strong biological activity. According to the statistics, over 60% of anticancer drugs are either directly or indirectly obtained from natural products [6].

What’s more, several reviews on BUC natural compounds have been published previously, but most cover only a limited number of natural products or focus on mechanisms, necessitating a more comprehensive and innovative analysis [7,8,9]. This review employs a systematic, chemically oriented classification framework, categorizing natural products primarily into terpenoids, phenolics, alkaloids, and other groups for discussion. This classification principle facilitates a comparative overview of research progress across different categories of natural products in bladder cancer therapy. By analyzing the antitumor mechanisms, pharmacodynamic characteristics, and research stages of representative compounds within each category, this framework clearly reveals the depth and maturity of research across different classes. This structured overview not only provides researchers with an intuitive map of progress but also offers an objective basis for prioritizing future resource allocation and research direction selection. Simultaneously, systematic coverage by chemical category facilitates a comprehensive examination of the polypharmacological potential inherent in natural products, avoiding omissions caused by single-target classification. This approach outlines a more comprehensive and systematic landscape of potential candidates for drug discovery based on natural product skeletons and the exploration of multi-target synergistic mechanisms. Crucially, this review systematically synthesizes preclinical evidence while intensifying the integration and evaluation of clinical research data related to natural products, updating progress in clinical studies. This focus significantly enhances the review’s translational medicine value. In-depth analysis of this section directly bridges critical gaps in translation from basic research discoveries to clinical practice, providing evidence-based information with direct reference value for clinical treatment, which advances natural products from laboratory research toward substantive progress in clinical adjuvant therapy for bladder cancer.

## 2. Terpenoids

In the strict sense, terpenes refer to a series of isomeric unsaturated hydrocarbons with the general formula C_10_H_16_, found in volatile *essential oils* (ethers) derived from plants. However, it was later realized that “terpenes” are simply a family of natural compounds formed by multiples of the C_5_H_8_ (isoprene) unit. Therefore, the meaning of “terpenes” was expanded to include some new, more distantly related compounds [10]. Existing research has demonstrated that terpenoids not only play a role in resisting foreign substances and facilitating communication between the same or different organisms in nature [11] but also exhibit significant antitumor [12,13,14,15,16], anti-inflammatory [17], anti-apoptotic [18,19,20], antibacterial [21], and antioxidant properties [22].

### 2.1. Potential of Monoterpenes in BUC Treatment

Monoterpenes are a major class of terpenoids, 10-carbon compounds formed by two connected isoprene units. Due to their low molecular weight, most monoterpenes exist in nature as *essential oils* [23].

#### 2.1.1. *Essential Oil*

Saini, D et al. [24] have shown by experiment that the components of *essential oils* cause ROS to be produced inside cells, thus damaging mitochondria and lowering the levels of mitochondrial membrane potential (ΔΨm), which then activates the mitochondrial-dependent endogenous apoptosis pathway [25]. Moreover, they found that *essential oils* could reduce MMP-9 levels to some degree, indicating that they may also inhibit cell migration.

#### 2.1.2. *Limonene* and *Genipin*

Apart from the *essential oil*, Motie FM et al. [26] combined *limonene* and cisplatin for treating the BUCs line 5637, the result showed that compared to cells treated by cisplatin or *limonene* separately, the combination of both reduced the activation of the PI3K/AKT/mTOR pathway, which increased the rate of apoptosis and inhibited cell activity.

What’s more, one in vivo study done on C57BL/6J male mice showed that *Genipin* helps stop cisplatin from hurting kidney tissues by lowering oxidative/nitrosative stress and inflammation, stopping cell death pathways, showing strong protection for the kidneys [27].

Research about monoterpenes can be found easily, but it’s still quite limited. Perhaps we will continue to do more in-depth studies on monoterpenes, such as *essential oils* or *limonene*, in the next phase of our work (Table 1).

### 2.2. The Potential of Sesquiterpenes in the Treatment of BUC

Sesquiterpenes are 15-carbon compounds composed of three isoprene building blocks [28], widely distributed in various angiosperms, some gymnosperms, and most plants [29]. In studies targeting BUC in this area, the most extensively studied compounds are *Artesunate* (ART) and *Atractylenolide* (ATR).

#### 2.2.1. *Artesunate*

ART is a water-soluble derivative of artemisinin, a first-line drug for malaria treatment [30], and earlier studies have shown that artemisinin also exhibits antitumor activity [31]. In studies investigating the efficacy of ART in BUC, Zuo W et al. [32] found that ART can enhance the expression of miR-16 protein in T24 cells, thereby inhibiting the expression of Cyclooxygenase-2 downstream, leading to reduced Prostaglandin E protein expression, and thus promoting tumor cell apoptosis. And more importantly, this effect exhibits strong selectivity and low toxicity toward normal human urothelial cells. In another study by Zhou X et al. [33], it was mentioned that ART can upregulate ROS and activate the AMPK-mTOR-ULK1 pathway in human bladder urothelial carcinoma cells (BUCs), thereby inhibiting the synthesis of Bcl-2 protein and promoting the synthesis of cleaved-caspase3 and cleaved-PARP proteins, ultimately inducing caspase-dependent apoptosis through autophagy. What’s more, another study on cisplatin-resistant BUCs, Zhao F et al. [34], reported that, at low concentrations (2.5 μM), ART blocks G1/S transition, triggering G0/G1 cell cycle arrest. At higher concentrations, ART (10 μM) induces mitochondrial dysfunction, activates autophagy, and mediates autophagy-dependent apoptosis in both cisplatin-sensitive and cisplatin-resistant BUCs. Therefore, ART holds promise as an adjuvant to improve cisplatin therapy in advanced BUC and cisplatin-resistant BUC patients. In addition to ART, in the study by Botrous S et al. [35], the well-known antimalarial drug artemisinin, when administered as a pretreatment before cisplatin therapy, not only enhances the efficacy of cisplatin in high-grade urinary tract epithelial cancer in male albino mice through the upregulation of FGFR3, HRAS, P53, and KDM6A but also significantly reduces cisplatin-induced kidney injury.

ART is a water-soluble derivative of artemisinin, which is a first-line drug for treating malaria [30], and previous research has demonstrated that artemisinin possesses antitumor properties [31]. Zuo W et al. [32] found in their studies on the effectiveness of ART in BUC that ART could increase the expression of miR-16 protein in T24 cells, which would inhibit the expression of Cyclooxygenase-2 downstream, causing a reduction in Prostaglandin E protein expression and thus promoting tumor cell apoptosis. Moreover, this effect shows great selectivity and little toxicity towards regular human urothelial cells. Another study from Zhou X et al. [33] said that ART can increase ROS and activate AMPK-mTOR-ULK1 pathway in human bladder urothelial carcinoma cells (BUCs), so it downregulates Bcl-2 protein and upregulates cleaved-caspase3 and cleaved-PARP proteins, which leads to apoptosis because of caspase-dependent autophagy. In another study about cisplatin-resistant BUCs, Zhao F et al. [34] reported that ART blocks G1/S transition and causes G0/G1 cell cycle arrest at low concentration (2.5 μM), and, at higher concentrations, ART (10 μM) induces mitochondrial dysfunction, activates autophagy, mediating autophagy-dependent apoptosis in both cisplatin-sensitive and cisplatin-resistant BUCs. Thus, ART looks hopeful as an add-on to make cisplatin work better for late-stage BUC and cisplatin-not-working-BUC patients. Besides ART, according to the study by Botrous S et al. [35], the famous antimalarial medicine artemisinin, if given as a pre-treatment before cisplatin treatment, can improve the efficiency of cisplatin on high-grade urinary tract epithelial cancer in male white mice via increasing FGFR3, HRAS, P53, and KDM6A, and also considerably lessen cisplatin-caused kidney damage.

#### 2.2.2. *Atractylenolide* (ATR)

ATR (-I, -II, and -III) are sesquiterpene compounds extracted from the traditional Chinese medicine *Atractylodes macrocephala Koidz*, and previous studies have confirmed the potential of ATR in anticancer activity [36]. In the treatment of BUC, Rui Yu et al. [37] experimentally demonstrated that ATR-1 upregulates p21 and downregulates cyclin B1, Cyclin-dependent kinase 1(CDK1), and Cdc25c, thereby arresting the cell cycle at the G2/M phase. Furthermore, ATR-1 triggers apoptosis through the activation of the mitochondrial apoptosis pathway, and the occurrence of apoptosis also depends on the inhibition of the PI3K/Akt/mTOR signaling pathway, leading to a further reduction in the expression of apoptosis-related proteins Bax and Bad. Finally, mouse studies demonstrated that ATR-1 blocked T-24 or 253J-induced xenograft tumor growth, and the good news is that the animals exhibited good tolerability.

#### 2.2.3. *Other Sesquiterpenes*

In addition to the aforementioned sesquiterpenes, the remaining sesquiterpenes shall be discussed in relation to the primary pathways inhibiting tumour growth, including: inducing BUC apoptosis, inhibiting the migration and invasion of BUCs, inhibiting the cell cycle of BUCs, and promoting the efficacy of traditional drugs.

If it comes to inducing BUC apoptosis, the following sesquiterpenes may come into view. Zhou L et al. [38] show that *curcumol*, extracted from the rhizome of C.aeruginosae [39], induces apoptosis in BUCs in a concentration-dependent manner by inhibiting the expression of enhancer of zeste homolog 2, a key regulator of cell survival and death. Specifically, intracellular ROS accumulation leads to the loss of ΔΨm, which in turn triggers downstream mitochondrial-dependent apoptosis. What’s more, *(-)-Gochnatiolide B*, a guaiacolide-type sesquiterpenoid dimer first isolated from the roots of *Gochnatia pomculata*, on the other hand, can induce cell apoptosis by increasing the protein levels of p21 and p27, leading to G1 phase arrest and subsequent caspase-dependent apoptosis [40]. For *cis-Nerolidol* [41], an aliphatic sesquiterpene commonly found in *essential oils* from plants with a floral odor [42], two different pathways induce apoptosis: in the early stages of *cis-Nerolidol* treatment, the signal is primarily transmitted through β-adrenergic receptors, PKA, and soluble adenylyl cyclase, leading to increased transport of calcium ions from the endoplasmic reticulum (ER) to the cytoplasm via ryanodine receptors. The increased calcium ion concentration in the cytoplasm induces ER stress, and the downstream p38-MAPK pathway is activated, leading to caspase-1-dependent paraptosis. In the later stages of treatment, prolonged high Ca^2+^ concentrations in the cytoplasm further induce ROS production, leading to prolonged activation of the downstream Extracellular-regulated kinase (ERK) pathway and ultimately triggering paraptosis. Apart from these, *Scabertopin scaber* L. [43] mentioned above can also activate RIP1/RIP3/MLKL phosphorylation by reducing the ΔΨm and stimulating mitochondrial ROS production, thereby mediating the occurrence of necroptosis in J82 cells.

When we are talking about the inhibition of the migration and invasion of BUCs, we will be talking about the following substances. First of all, *Scabertopin scaber* L. [43], one of the major sesquiterpene lactones found in *Elephantopus scaber* L., mediates necrotic apoptosis by inducing ROS in BUCs in vitro and inhibits MMP-9 expression by suppressing the FAK/PI3K/Akt signaling pathway, ultimately inhibiting the migration and invasion capabilities of BUCs. Apart from the *Scabertopin scaber* L., *antrocin* [44] inhibits the migration and invasion of human BUCs at non-cytotoxic concentrations by suppressing the ERK/FAK/p21 and ERK/c-Fos/MMP-2 signaling pathways. At cytotoxic concentrations, *antrocin* induces apoptosis in human BUCs through both exogenous and endogenous signaling pathways, as evidenced by the upregulation of proteins such as Fas, DR5, and Bax [34,43,44].

What’s more, in terms of inhibiting the cell cycle of BUCs, *Scabertopin scaber* L. [43] induces a significant increase in the percentage of S and G2/M phase cells in a concentration-dependent manner. Moreover, following treatment with *cis-Nerolidol* [41], BUCs exhibit G1 phase accumulation, and all of these produce an inhibitory effect on BUC proliferation.

In addition to the synergistic effects mentioned above, when combined with traditional chemotherapy drugs, *Mogoltacin* [45], a sesquiterpenoid coumarin derived from *Ferula badrakema*, enhances the cytotoxicity of the traditional chemotherapy drug vincristine in human transitional cell carcinoma (TCC) cell lines (Table 2).

### 2.3. Diterpenes in the Treatment of BUC

Diterpenes are composed of two monoterpene units, i.e., 20-carbon compounds formed by four isoprene building blocks, which are secondary metabolites of terrestrial and marine organisms [47].

#### 2.3.1. *Paclitaxel*

When studying diterpenoids, one that cannot be overlooked is *paclitaxel*, a tetrahydroditerpenoid compound [48], discovered by Monroe Wall and Mansukh Wani in 1963 from the Pacific yew tree Taxus baccata [49]. It has been widely used in the treatment of ovarian cancer, breast cancer, uterine cancer, and so on [50].

In existing research on bladder cancer, K Inoue et al. [51] proposed that the anti-epidermal growth factor receptor monoclonal antibody C225 interacts with *paclitaxel*. C225 can downregulate Raf-1 kinase activity, thereby enhancing *paclitaxel*-induced cytotoxicity. Additionally, *paclitaxel* can increase C225’s anti-angiogenic and tumor cell metastasis capabilities by downregulating the expression of basic fibroblast growth factor, vascular endothelial growth factor (VEGF), IL-8, and MMP-9. In the same year, K Inoue et al. [52] reported that the combination of *paclitaxel* with another drug, the anti-vascular endothelial growth factor receptor monoclonal antibody DC101, demonstrated a higher apoptosis rate in endothelial cells and tumor cells. Similarly, Keiji Inoue et al. [53] proposed three years later that docetaxel enhances the therapeutic effect of the angiogenesis inhibitor TNP-470 (AGM-1470) in metastatic human transitional cell carcinoma, with this effect at least partially achieved through inhibition of angiogenesis, bFGF, and MMP-9 expression, as well as induction of apoptosis.

But clinically, *paclitaxel* is still not widely used as the therapeutic schedule, thus the existing studies in this field mainly focus on clinical transitional studies investigating the combination of *paclitaxel* with other chemotherapeutic agents, intending to overcome the issue of drug resistance to traditional chemotherapy drugs, including studies on extremely high-risk NMIBC [54], preoperative MIBC patients [55], HER2-positive recurrent or metastatic BUC [56], MIBC in non-cystectomy candidates [57], post-operative MIBC [58], BCG-unresponsive NMIBC [59], cisplatin therapy for advanced BUC [60], and unspecified advanced BUC [61]. The results of all these studies demonstrate good drug tolerability and lower adverse reaction rates compared to monotherapy, providing new treatment options and approaches for refractory, recurrent, metastatic, and MIBC (Table A1).

#### 2.3.2. *Jolkinolide B*

Apart from *paclitaxel*, research in the field of BUC treatment has also repeatedly mentioned a compound derived from the traditional Chinese medicine *Euphorbia fischeriana* Steud., *Jolkinolide B* (JB), which has garnered increasing attention due to its potent antitumor activity. Jun Sang et al. published three studies on JB for BUC treatment in 2021, 2022, and 2024. In the 2021 study [62], it was mentioned that JB targets the thioredoxin and glutathione systems in BUCs, inhibiting Thioredoxin Reductase 1 (TrxR1) and depleting GSH, thereby inducing ROS production. Excessive ROS then upregulates the expression of p-JNK, p-p38, and p-ERK in T24 and UM-UC-3 cells, thereby activating the MAPK/ERK pathway and inducing paraptosis in tumor cells. Additionally, excessive ROS production induces ER stress, further exacerbating paraptosis. Beyond paraptosis, the overexpression of p-JNK, p-p38, and p-ERK induced by ROS also triggers cell apoptosis, thereby achieving the therapeutic objective. Furthermore, the in vivo studies in this experiment also demonstrated significant anti-BUC activity and good tolerability. In subsequent research in 2022 [63], Jun Sang et al. conducted studies on mammalian target of rapamycin (mTOR) inhibitors, which have been demonstrated they lead to Akt feedback activation and the occurrence of protective autophagy, resulting in the development of drug resistance in BUCs. However, Jun Sang et al. found that JB can enhance BUC sensitivity to mTOR inhibitors (mTORi) by dual inhibition of Akt signaling and autophagy. Furthermore, they experimentally demonstrated that mTORi can also enhance the effects of JB in inducing cell paraptosis and apoptosis through the ER-stress and MAPKs pathways, thereby enhancing the sensitivity of BUCs to mTORi. In 2024 [64], Jun Sang et al. experimentally confirmed that JB can also synergistically enhance the antitumor activity of Glutathione peroxidase 4 (GPX4) inhibitors by inhibiting TrxR1 in cisplatin-resistant BUCs, further inducing ferroptosis and apoptosis in cisplatin-resistant BUCs through lipid reactive oxygen species. The increased expression of TrxR1 was proven to be the cause of cisplatin resistance in BUCs.

#### 2.3.3. *Abietic Acid*

In addition to the two major classes of substances extensively studied in BUC therapy, Yi Xu et al. [65] proposed that *Abietic acid* (AA), an abietane diterpene isolated from *Pimenta racemosa* var. *grissea*, can activate the heme oxygenase-1 (HO-1) pathway in BUCs, thereby inhibiting Glutathione peroxidase 4 (GPX4), leading to ferroptosis in BUCs. Furthermore, they demonstrated through in vivo experiments that AA exhibits high selectivity and good drug tolerance. In experiments combining AA with other traditional chemotherapy drugs, they also found that AA significantly increases cell death induced by cisplatin, *paclitaxel*, gemcitabine, and gefitinib.

#### 2.3.4. *Other Diterpenes*

The anti-tumor properties of other diterpenes will also be described according to some major anti-tumor pathways, including inhibiting cell cycle stagnation and regulating the tumor microenvironment.

In the field of inhibiting cell cycle stagnation, Siwen Niu et al. [66] utilized new aphidicolin diterpenoid compounds derived from the deep-sea-derived fungus *Botryotinia fuckeliana* to conduct in vitro experiments on BUCs. Among the 37 new non-bisphenol derivatives extracted, compound 32 exhibits the most pronounced cytotoxic effect against T24 cells, primarily by inhibiting T24 cell migration and blocking T24 cells in the G0/G1 phase by reducing the protein levels of CDK4, CDK6, and cyclin D1 in T24 cells, thereby achieving an anti-T24 cell effect.

Apart from the mechanisms mentioned above, some research on the tumor microenvironment has been conducted. First of all, Shuangjie Liu et al. [67] conducted mouse model experiments using a biologically active endo-beta-oligomeric diterpene *Oridonin* [68], which demonstrates that *Oridonin* inhibits BUC glycolysis by covalently targeting and binding to Hexokinase I, thereby suppressing Warburg effect-mediated BUC survival and immune evasion. Furthermore, the study finds that *Oridonin* combined with PD-L1 inhibitors alleviates immune suppression and increases the content of cytokines secreted by CD8+ T cells, thereby enhancing the antitumor effects of the tumor immune microenvironment. In another study on the tumor microenvironment, Alexander T H Wu et al. [69] demonstrated that *Ovatodiolide*, isolated from *Anisomeles indica*, inhibits bladder carcinogenesis by suppressing mTOR/β-catenin/CDK6 signaling and exosome-mediated oncomiR released from M2 tumor-associated macrophages. Furthermore, *Ovatodiolide* demonstrates superior therapeutic efficacy against cisplatin-resistant BUCs in combination experiments (Table 3).

### 2.4. Potential of Triterpenes in BUC Treatment

Triterpenes are a type of terpenoid compound, consisting of 30 carbon atoms formed by the condensation of six isoprene units. They exist in plants in free form, as glycosides or esters bound to sugars [70]. In the field of BUC treatment, the most extensively studied triterpenes are *Betulinic acid* (BA), followed by *Asiaticoside*.

#### 2.4.1. *Betulinic Acid*

BA is a pentacyclic triterpenoid compound [71] found in the bark of birch trees. Young Kim et al. [72] utilized it to perform a series of in vitro experiments on human BUC lines T24, UMUC-3, and 5637. The results show that BA causes a decrease in the expression of Bcl-2 and an increase in the levels of Bax in BUCs lines, further inducing the activation of the endogenous mitochondrial-dependent apoptosis pathway. Additionally, the experiments further demonstrated that BA also induces apoptosis in human BUCs by downregulating the expression of G2/M-related proteins, which is partially due to G2/M phase arrest. In the final of the experiment, they demonstrated that, after treatment with BA, reduced levels of MMP-9 were detected in all these cell lines, which hints that BA may inhibit the migration and invasion of BUCs. What’s more, in the study by Yan Zhang et al. [73], they conducted corresponding in vivo and in vitro experiments, suggesting that BA induces autophagy-dependent apoptosis in human BUCs through the Bmi-1/ROS/AMPK-mTOR-ULK1 axis, and also demonstrated significant therapeutic effects in the experiments on mice.

#### 2.4.2. *Asiaticoside*

*Asiaticoside* (*AC*) is a triterpene saponin derived from *Centella asiatica* [74]. Ming Jin et al. [75] investigated it in combination with propofol, based on the previously established ability of propofol to regulate the malignant progression of BUC, including proliferation, apoptosis, stem cell-like properties, migration, and invasion [76,77], and then they investigated the specific effects of the triterpenoid derivative AC in combination with propofol. The result shows that after combination therapy, the levels of N-cadherin decrease and E-cadherin increase significantly in BUCs. Intracellular Fe^2+^ level increases, ROS level rises, and the expression of solute carrier family 7 member 11 (SLC7A11) and GPX4 decreases to a greater extent than when propofol was used alone. The percentage of CD8+ T cells, the concentration of interferon-γ, and the expression level of PD-L1 in BUCs also increases to a greater extent. The above findings indicate that the addition of AC enhances the ability of propofol to inhibit the epithelial–mesenchymal transition (EMT) in BUC, induce iron death in BUCs, and suppress tumor immune escape, demonstrating a synergistic effect. Additionally, the experiment demonstrates that the addition of AC further enhances propofol’s inhibitory effect on the PI3K/AKT pathway expression.

#### 2.4.3. *Other Triterpenes*

The same as before, the antitumor effects of the other triterpenes will also be conducted into several categories, including inhibiting BUC apoptosis, inhibiting BUC metastasis and invasion, and regulating the tumor immune microenvironment.

First, when we are talking about the induction of BUCs apoptosis, these triterpenes may come into our mind: *Maslinic acid*, *Pachymic acid*, *β-amyrin*, and *Brusatol*. First of all, in the study of Shilong Zhang et al. [78], *Maslinic acid* can induce BUC apoptosis by activating the p38 MAPK signaling pathway. Additionally, they confirmed in animal experiments that this apoptotic effect of *Maslinic acid* exhibits significant selectivity. Secondly, Jin-Woo Jeong et al. [79] demonstrate that *Pachymic acid* induces caspase-dependent apoptosis by upregulating the exogenous TNF-related apoptosis-inducing ligand (TRAIL) receptor pathway, and further induces related endogenous apoptosis pathways by regulating Bcl-2 and inhibitor of apoptosis protein family members in EJ BUCs. What’s more, Kai-Wei Lin et al. [80] find out that *β-amyrin* can lead to ROS accumulation in NTUB1 cells, leading to cell cycle arrest and triggering apoptosis. But when it comes to *Brusatol*, the biological effects it causes will be a little different. Xi Yu et al. [81] found that *Brusatol* disrupts the redox homeostasis in BUCs by inhibiting the Chac1/Nrf2/SLC7A11 pathway: decreasing GPX4 levels, accumulation of intracellular ROS and Fe^2+^, and subsequent induction of ferroptosis in the experimental cells. In addition to the aforementioned substances being extensively studied, Ruizhen Ru et al. [82] find that the sea cucumber triterpenoid *Frondoside A* can induce apoptosis independent of caspase and TP53, and the result of the research is still at a superficial level.

In terms of inhibiting BUC metastasis and invasion, Dayin Chen et al. [83] conducted a series of in vitro cell and in vivo animal experiments using *Platycodin D* (PD), the major saponin extracted from *Platycodonis radix*. Their findings suggest that PD can downregulate LncRNA-XIST, promote the expression of miR335, thereby inhibiting the development of BUC malignant phenotypes, and promote BUCs apoptosis in a dose- and time-dependent manner. Ruizhen Ru et al. [82] also confirmed through in vivo and in vitro experiments that the sea cucumber triterpenoid *Frondoside A* can inhibit cell viability and migration, but the specific mechanisms remain unclear.

In terms of regulating the tumor immune microenvironment, Chenfan Kong et al. [84] started with novel materials. They conducted a series of in vitro and in vivo studies using tumor-targeted nanomicelles loaded with *Astragaloside IV*, a widely used Chinese herbal medicine derived from *Astragali Radix* [85]. The results indicate that *Astragaloside IV* exerts a potent dual inhibitory effect on STAT3 and NF-κB in tumor cells MB-49, thereby inhibiting tumor angiogenesis. The results also demonstrate that this complex promotes the infiltration and activation of CD8+ T cells while reducing the infiltration of regulatory T cells in tumors, a finding further validated in subsequent studies combining it with aPD-L1. Additionally, during the experiments, the nanocomplex exhibits excellent targeting efficacy, with the drug complex accumulating significantly more in bladder tumors than in the lungs or liver (Table 4).

### 2.5. Tetraterpenes and Tetraterpenoid Compounds (Carotenoids)

Tetraterpenes are the last subclass within the terpenoid compound family, consisting of 40-carbon compounds formed by the condensation of eight isoprene units [87]. They are primarily divided into two categories: carotenoids and oxygenated carotenoids, derived from plants, fungi, and bacteria, which the animals cannot synthesize and generally obtain through diet [88].

**Figure 1:**
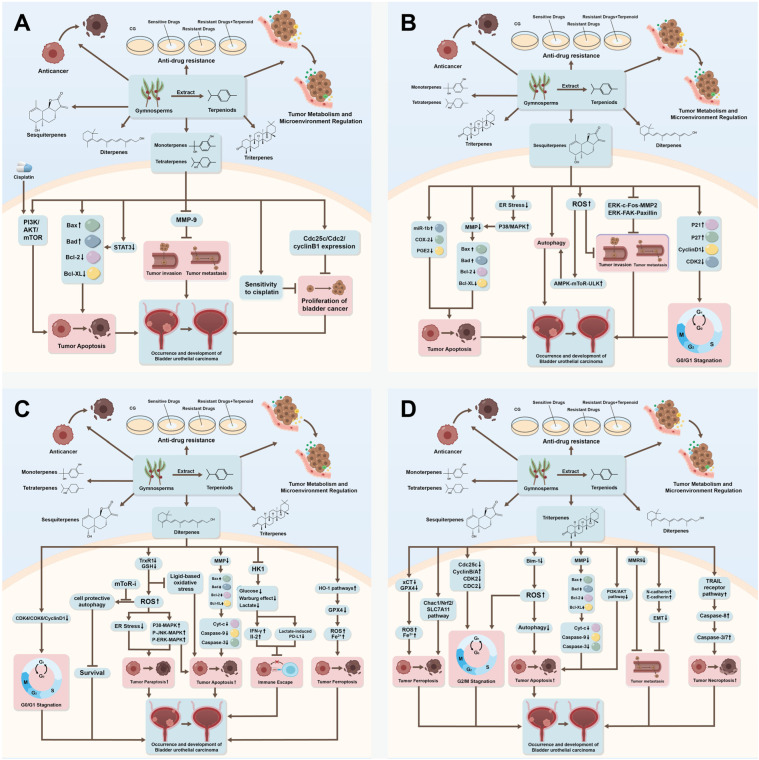
(**A**) The summary of anticancer effects and the mechanism of Monoterpenes and Tetraterpenes action. Monoterpenes may modulate numerous signaling pathways, including apoptosis, metastasis, as well as cell cycle. And Tetraterpenes mainly focus on activating Apoptosis and increasing the cell’s sensitivity to cisplatin. (**B**) The summary of anticancer effects and the mechanism of Sesquiterpenes’ action. Sesquiterpenes may play a role in anti-bladder cancer by activating apoptosis, paraptosis and necroptosis; inhibiting migration and invasion; inducing cell cycle stagnation and the growth of bladder cancer cells. Moreover, they can also enhance tumor cells’ sensitivity to cisplatin and the traditional drugs. (**C**) The summary of anticancer effects and the mechanism of Diterpenes’ action. Diterpenes mainly produce the effects of activating Apoptosis, paraptosis, and Ferroptosis; inducing cell cycle stagnation; inhibiting the malignancy of bladder cancer cells; inhibiting tumor growth and metastasis; enhancing traditional drug-induced cytotoxicity; lowering cell’s resistance to traditional drug and what’s more, inhibiting the immune escape and changing the Metabolic type of bladder cancer cells. (**D**) The summary of anticancer effects and the mechanism of Triterpenes’ action. Triterpenes may modulate numerous signaling pathways, thereby leading to many anti-bladder cancer effects, including activating Apoptosis, Necroptosis, Ferroptosis; resulting in cell cycle stagnation; enhancing cytotoxic effect of the traditional drug like cisplatin; inhibiting the Metastasis of bladder cancer cells; inhibiting tumor angiogenesis and regulating the tumor immune microenvironment. Generated using Adobe Illustrator (Version 26.3).

However, research on Tetraterpenes in BUC treatment appears to be limited. Wen-Chyi Dai et al. [89] conducted a series of in vitro studies using a Marine-Derived Carotenoid, *Fucoxanthin*, on human bladder TCC cell lines. The results suggest that *Fucoxanthin* inhibits the expression of STAT3 in TCC cell lines, thereby downregulating Bcl-xL expression and increasing the sensitivity of TCC cell lines to cisplatin. However, the limitations of the experiment include the fact that it was only conducted in vitro on cells, and the mechanism by which *Fucoxanthin* leads to a decrease in STAT3 expression remains unclear. Additionally, Jung Park et al. [90] conducted a meta-analysis on the effects of vitamin and antioxidant supplements on the prevention of BUC, concluding that vitamin or antioxidant supplements have no preventive effect on BUC (Figure 1) (Table 5).

## 3. Phenols

Phenolic natural products show great promise as anti-tumor agents because they come from many different living things and have lots of different chemical forms. Some studies have shown that they can fight cancer by stopping tumors from growing, making cells die, and changing how the body’s defenses work. This section talks about some phenolic compounds’ roles in bladder tumors and whether they could become useful one day.

### 3.1. Curcumin

*Curcumin* is an orange-yellow natural substance that comes from *turmeric rhizomes*, and it’s one kind of *polyphenol* that can prevent inflammation and stop oxidation. Moreover, it has also been shown to have a significant therapeutic effect on cancer, age-related diseases, and autoimmune diseases [91,92,93]. And among them, *curcumin* showed multi-dimensional anti-tumor effects for BUC treatment.

*Curcumin* has an anticancer effect by stopping the growth of cancer cells and causing cell death. Many studies show that the transmembrane receptor Trop-2 on the surface of trophoblasts is important for tumor growth and spread, so it’s a good target for treatment. IGF-2/IGF-1R signaling axis maintains CSC stemness and radiation resistance by PI3K positive feedback loop; HER-2 overexpression drives invasive growth via MAPK/PI3K pathway. These three parts make up a multi-dimensional pro-cancer network that has a big part to play in how tumors grow and how people do after getting sick. Targeted intervention can synergistically inhibit drug resistance and recurrence and enhance the sensitivity to radiotherapy and chemotherapy [94,95,96,97]. Zhang et al. showed that *curcumin* could significantly decrease the expression of Trop2 and its downstream target cell cycle protein E1, raise the amount of p27, and stop the growth, movement, and spread of BUCs [98]. IGF-2 (insulin-like growth factor 2) promotes cancer stem cell self-renewal and drug resistance through a positive feedback loop of PI3K signaling, while IGF-1R (Insulin-like growth factor-1 receptor) signaling upregulation results in resistance to radiotherapy, and targeted inhibition of this pathway synergistically improves radiotherapy sensitivity and inhibits tumor recurrence. HER-2 is overexpressed in many cancers. It promotes cell growth and is linked to the development of invasive tumors and unfavorable health outcomes. *Curcumin* and its derivatives display antitumor activity by suppressing the expression of IGF-2 and HER-2 and blocking the PI3K/AKT/mTOR signaling pathway, which inhibits the proliferation of BUCs and induces apoptosis [99,100]. Gao et al. found that *curcumin* promotes the ubiquitin-proteasome degradation of the oncogenic transcription factor Krüppel-like factor 5 by inhibiting the expression of YAP/TAZ in BUCs, leading to a reduction in cyclin D1 expression, thus inhibiting tumor cell proliferation and promoting apoptosis [101].

In terms of anti-metastatic effects, *curcumin* protects the integrity of the basement membrane by inhibiting matrix metalloproteinases (MMPs), blocking the ability of tumor cells to penetrate the basement membrane [102]. Moreover, *curcumin* reversed aniline-induced EMT via the ERK5/AP-1 pathway, causing an increase in E-cadherin expression and a decrease in Vimentin expression, thus inhibiting cancer cell invasion [103]. In a tobacco-induced BUC model, *curcumin* reversed chronic tobacco smoke exposure-induced EMT and cancer stem cell properties in the bladder by inhibiting the Wnt/β-catenin signaling pathway and decreasing the expression of EMT-related markers, N-cadherin and Vimentin, thus inhibiting the malignant progression of BUC [104].

*Curcumin* also plays an important role in overcoming drug resistance in BUC. BCG, an adjuvant intravesical immunotherapeutic agent for NMIBC, can effectively reduce the recurrence of BUC. However, there are still a high number of patients who recur or do not even respond to BCG [105]. *Curcumin* inhibits BCG treatment-induced NF-κB activation and upregulates TRAIL receptor expression, synergistically enhancing the antitumor effects of BCG in BUC [106]. Gupta et al. showed that *curcumin*-dichloroacetic acid hybrid molecules could effectively suppress the clonal survival ability of BUC drug-resistant cells, including multidrug-resistant cells resistant to cisplatin, gemcitabine, and combination chemotherapy. These molecules show promise in breaking through the chemotherapy resistance barrier in both short-term and long-term treatments, giving a new way to stop the spread of MIBC [107]. Furthermore, *curcumin* selectively kills BUCs via a “synthetic lethality” mechanism that inhibits Aurora A kinase activity and induces autophagy, potentially circumventing traditional chemotherapy resistance pathways such as DNA repair or drug efflux, offering a novel approach to overcome cisplatin and other drug resistance. However, its efficacy in resistance models remains to be confirmed [108].

Although *curcumin* has multiple potentials such as anti-inflammatory, antioxidant, and anticancer, its low water solubility, rapid metabolism, and poor intestinal absorption lead to low bioavailability, which inevitably affects the effect [109]. Many studies have been done to find ways to make *curcumin* better absorbed by the body [110]. Xing et al. showed that pH-sensitive Zn(II)-*curcumin* nanoparticles can release *curcumin* and siRNA targeting oncogenic genes in the acidic tumor microenvironment, induce apoptosis, and inhibit the proliferation of BUCs, significantly reduce the tumor volume in vivo, and reduce the toxicity to normal tissues caused by pH sensitivity [111].

### 3.2. Flavonoids

#### 3.2.1. *Flavones*

In this section, we will review the function of five flavonoids: *apigenin*, *luteolin*, *baicalein*, *scutellarin*, *tangeretin*, and *chrysin* in BUC.

*Apigenin*, a natural flavonoid, has recently been shown to have multi-dimensional inhibitory effects on BUCs. *Apigenin* can block the progression of BUC T-24 cells into the G2/M phase by regulating cell cycle-related proteins, such as reducing cyclin B1 and CDK1 expression, and this cycle blocking effect is positively related to the dose, so it can inhibit the proliferation of tumor cells [112]. Additionally, *apigenin* increases the pro-apoptotic protein Bax and decreases the anti-apoptotic protein Bcl-2, causing a decrease in ΔΨm and activation of the caspase cascade reaction, resulting in programmed cell death of tumor cells, breaking the single path of DNA damage caused by traditional chemotherapy drugs [112]. In the inflammatory microenvironment, *apigenin* can significantly inhibit IL-1β-induced urokinase-type plasminogen activator receptor expression and inhibit AP-1 and NF-κB transcription factors’ activities through blocking the MAPK pathway’s phosphorylation, which can prevent tumor invasion and metastasis [113]. *Apigenin* works on the tumor cell cycle and apoptosis processes directly and also manages inflammatory signals within the tumor environment, hindering both the initial tumor development and the pathways related to metastasis, indicating significant therapeutic promise [114].

*Luteolin* is a natural flavonoid compound widely found in fruits and vegetables, with anti-inflammatory and anti-cancer bioactivities [115]. According to the research done by Iida et al., *luteolin* increases p21 in BUCs, causes G2/M phase arrest, reduces phosphorylated-mTOR and its downstream effector protein p-S6K, and blocks protein synthesis and tumor growth [116].

*Baicalein*, mainly obtained from the plant *Scutellaria baicalensis* of the Labiatae family, has antitumor effects on various tumors [117]. In BUC, *baicalein* can inhibit the proliferation, invasion of BUCs, and induce their apoptosis through blocking the cell cycle, inhibiting cell cycle proteins and matrix metalloproteinase activity, and activating the caspase-dependent apoptosis pathway [118]. *Baicalein* causes apoptosis in BUCs by activating the death receptor-mediated exogenous apoptotic pathway and the mitochondria-mediated endogenous apoptotic pathway, causing ROS-dependent disruption of the ΔΨm and the caspase-8/-9/-3 cascade, increasing the expression of pro-apoptotic protein Bax and decreasing the expression of anti-apoptotic protein Bcl-2 [119].

*Scutellarin* is the major active component obtained from *Scutellaria baicalensis* and other plants in traditional Chinese medicine. It has a broad spectrum of biological activity and shows good results in the prevention and treatment of cardiovascular diseases, tumors, diabetes, etc. [120]. BUC metastasis is associated with hypoxia-induced EMT, and *scutellarin* can block this process through the inhibition of PI3K/Akt and MAPK signaling pathways, reducing the migration, invasion, and metastasis of BUCs in vivo, with minimal toxicity to normal cells, making it a promising therapeutic approach for targeting tumor metastasis [121].

*Tangeretin*, a natural flavonoid, can greatly inhibit the proliferation of BUCs and cause apoptosis by affecting mitochondrial function. The mechanism of action of *tangeretin* includes lowering the ΔΨm, promoting cytochrome C (Cyt-c) release, regulating the imbalance of the Bax/Bcl-2 ratio, and activating the caspase-9/-3 cascade. Proteomics further revealed that it inhibited oxidative phosphorylation and increased ROS generation, disrupting energy metabolism. Also, *tangeretin* shows low toxicity to normal cells, and there is potential for it to be used as a targeted therapy for BUC [122].

#### 3.2.2. *Flavanols*

Catechins are a group of natural flavanols that can be found in abundance within *Camellia sinensis*, including *epigallocatechin gallate* (EGCG), *epicatechin*, *catechin*, and their derivatives as key parts. Catechins have considerable anticancer potential via multi-targeted modulation of oxidative stress, inflammation, epigenetic reprogramming, and metabolism [123,124]. In BUC, such polyphenolic substances provide new treatment options for reversing chemoresistance and preventing postoperative recurrence because of their multi-dimensional mode of action. The portion of this review talks about how EGCG works in BUC.

EGCG has become a subject of study because it has good redox regulation capabilities and can fight against tumors. Studies show that EGCG can stop the growth of BUCs in different ways at the molecular level, and it can greatly reduce how much BUC SW780 cells grow and move by lowering the NF-κB signaling pathway and its target gene, MMP-9 [125]. In addition, EGCG also induces autophagy-dependent apoptosis via inhibition of PI3K/AKT/mTOR pathway and significantly upregulates the expression of autophagy-related proteins, including LC3B II/Beclin, and inhibits the growth of BUCs [126]. EGCG inhibits BUC stem cells by specifically suppressing the expression of important proteins Gli1 and Ptch1 in the Sonic Hedgehog pathway, which blocks the self-renewal ability of tumor stem cells [127]. Resveratrol oligomers showed a synergistic effect with catechins on the cytotoxicity towards the BUC T24 cell line [128].

The therapeutic efficiency of catechins is further improved with the help of nanodelivery technology, and Chen et al.’s Mg (II)-catechin nanocarrier system can effectively deliver siRNA against eukaryotic translation initiation factor 5A2, which can greatly inhibit the growth of BUCs both in vitro and in vivo [129].

In short, catechins, particularly EGCG, hold much potential for BUC therapy via multi-pathway intervention and nanotechnology enhancement.

#### 3.2.3. *Chalcones*

*Isoliquiritigenin* (ISL), *licochalcone A* (LCA), and *echinatin* (ECN) are all chalcones, natural compounds discovered in licorice. The studies showed that ISL at 15 μM and 25 μM decreased the viability of BUC T24 cell line, cisplatin had a greater effect when combined with ISL, and ISL could antagonize the nephrotoxicity of cisplatin by regulating antioxidant proteins to protect normal renal cells [130]. LCA causes DNA damage by increasing the amount of reactive oxygen species, which stops the cell cycle at the G2/M stage and starts apoptosis through mitochondria [131]. ECN was observed to cause G2/M cell cycle arrest due to decreased cyclin B1 and CDK1 expression, along with inhibition of proliferation marker PCNA and v-mycmyelocytomatosis viral oncogene homolog. ECN also activates p38 kinase phosphorylation to promote apoptosis and enhances phosphorylation at the GSK-3β site, which inhibits the Wnt/β-catenin signaling pathway and promotes β-catenin degradation, thus preventing the malignant transformation of tumors. Moreover, it can reverse the epithelial–mesenchymal transition process, decrease the activity of matrix metalloproteinases, and greatly reduce the migratory and invasive capabilities of cancer cells. Furthermore, ECN has synergistic antitumor effects with cisplatin and gemcitabine in vitro and in vivo, and it is less toxic to normal cells, indicating that it may have clinical application prospects for the treatment of BUC in combination therapy [132].

#### 3.2.4. *Isoflavones*

*Genistein*, an isoflavonoid found in *soybeans*, plays an important anticancer role [133]. Wada et al. discovered that higher intakes of total soy isoflavones and their major components, daidzein and genistein, were associated with a reduced risk of BUC, particularly among males, according to the Takayama City Cohort Study on the relationship between dietary consumption of soy isoflavones and the risk of BUC [134]. *Genistein* was also shown to cause a G2/M phase block in BUC T24 cells. Mechanism: ROS-dependent inhibition of PI3K/Akt pathway results in upregulation of proapoptotic proteins (Bax) and downregulation of antiapoptotic proteins (Bcl-2), leading to mitochondria-dependent apoptosis [135]. *Formononetin* is the main active ingredient of Astragalus membranaceus. It has been shown that formononetin can inhibit the growth and invasion of cancer cells through the inhibition of PI3K/Akt signaling pathway. Zhou et al. used transcriptomics to show that it specifically targets tumor cells and that its antitumor effects are increased when combined with gemcitabine [136]. Other experiments have also shown that it can reverse the EMT process, for example, the upregulation of E-cadherin and the downregulation of N-cadherin, and inhibit the activity of matrix metalloproteinases, inhibit tumor invasion, and have low toxicity to normal uroepithelial cells [137].

#### 3.2.5. *Flavonols*

**Figure 2:**
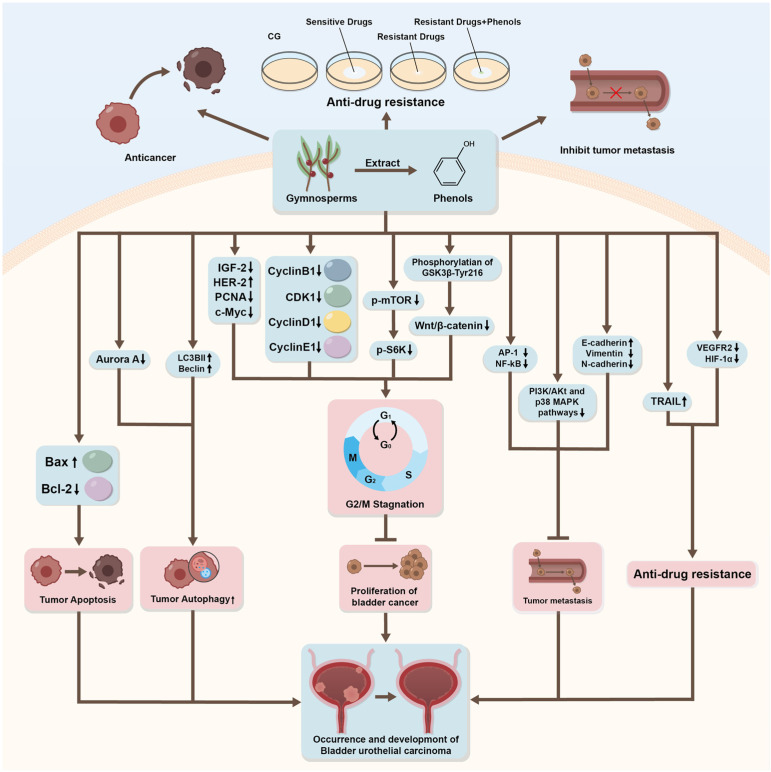
Summary of the anti-bladder cancer effects and mechanisms of action for several phenolic natural products. Summary of the anti-bladder cancer effects and mechanisms of action for several phenolic natural products. Phenolic compounds can modulate multiple signaling pathways, including inhibiting tumor deterioration, inhibiting tumor proliferation, anti-metastasis, and cancer cell apoptosis. Generated using Adobe Illustrator (Version 26.3).

*Quercetin* is a natural flavonol compound found in apples, tea, and red wine. Wu et al. demonstrated that *isoquercetin* (a quercetin glycoside derivative) causes apoptosis in BUCs via the AMPK signaling pathway, as indicated by the depolarization of ΔΨm, upregulation of the expression of the pro-apoptotic protein Bax, and activation of Caspase-3 [138]. The clinical application of *quercetin* is restricted due to its poor bioavailability; new nano-delivery systems offer a fresh approach to address the quercetin bioavailability issue [139,140,141]. *Quercetin*-coated titanate nanotubes created by Alban et al. not only enhanced the bioavailability of quercetin but also made BUC T24 cells more sensitive to in vitro radiotherapy [142] (Figure 2) (Table 6).

## 4. Alkaloids

Alkaloids represent a category of nitrogenous, commonly found in plants, displaying diverse biological activities. Emerging studies have demonstrated that numerous alkaloids exert BUCs effects via mechanisms including the regulation of tumor-associated signaling cascades, induction of apoptosis or autophagy, and suppression of cell proliferation and metastasis. This part comprehensively summarizes the alkaloids that have been intensively investigated, while briefly outlining other related compounds based on their anti-BUC mechanisms of action.

### 4.1. Berberine

*Berberine* shows similar functions as HER2 inhibitors through decreasing the expression of HER2/PI3K/AKT signaling proteins, thus reducing the proliferation, migration, invasion, and cell cycle progression of BUCs and promoting their apoptosis [143]. Inactivation of the PI3K/AKT pathway by *Berberine* results in decreased expression of Rad51, thereby enhancing the cytotoxicity, apoptosis induction, and migration suppression effects of gemcitabine on BUCs, along with a reduction in Akt phosphorylation induced by gemcitabine [144]. At the epigenetic level, *Berberine* increases miR-17-5p, which directly binds to the 3′UTR of JAK1 and STAT3, leading to a decrease in the expression of both proteins, blocking the JAK1-STAT3 signal transduction pathway, and finally achieving an anti-BUC effect [145]. *Berberine* causes S phase cell cycle arrest through a synergistic mechanism that involves increasing P21 and P27 protein expression and lowering Cyclin D, Cyclin A2, and CDK2 protein expression. It does more than stop EMT, which rearranges the cytoskeleton to stop BUC spreading. Also, *Berberine* reduces the expression of antioxidant-related genes such as Nrf2, HO-1, superoxide dismutase, and GPX1, raising the level of intracellular ROS. Accumulation of ROS increases the Bax/Bcl-2 ratio, which triggers the intrinsic apoptotic pathway. Additionally, ROS accumulation negatively regulates the NF-κB pathway, which is one of its antitumor activities [146].

### 4.2. Capsaicin

*Capsaicin* reduces BUC growth and induces apoptosis by reducing the expression of tumor-associated NADH oxidase and reducing the expression of several proteins related to the cell cycle, leading to prolonged cell doubling time and cell cycle arrest. Also, *Capsaicin* inhibits ERK activation and reduces the phosphorylation of p21 and FAK, which leads to a decrease in cell migration [147]. Moreover, *Capsaicin* acts as a Transient Receptor Potential Vanilloid 1 (TRPV1) agonist that causes growth arrest in TRPV1-overexpressing BUCs and improves the anticancer effects of pyruvate by inducing S-phase arrest [148].

### 4.3. Liensinine (LIEN)

In BUC treatment, *LIEN* induces cellular senescence and suppresses proliferation by inhibiting the CDK2/4 and PI3K/AKT pathways in BUCs, and the PI3K/AKT signaling pathway is critical for LIEN’s anti-BUC activity that LIEN inhibits tumor growth by reducing the expression of phosphorylated AKT1 (p-AKT1) and promoting AKT1 degradation, thereby blocking the PI3K/AKT signaling cascade and decreasing p-AKT levels to activate the FOXO3a signaling pathway [149].

### 4.4. Matrine (MAT)

*MAT* exerts no adverse effects on the growth of normal urinary tract epithelial cell lines, but it suppresses the growth, invasion, and migration of BUCs. Its anti-BUC mechanism involves upregulating the expression of LINC00472/PDCD4 and blocking the PTEN/PI3K/AKT pathway in BUCs [150]. Furthermore, *MAT* reduces NOX2 expression levels and inhibits the transformation of bladder epithelial cells [151]. *MAT* suppresses BUC proliferation, induces apoptosis, and causes cell cycle arrest by abnormally activating the PI3K/AKT/FoxO3a signaling pathway. Additionally, it exerts a growth-inhibitory effect on BUCs by promoting intracellular ROS production and activating the mitochondrial ROS-mediated signaling pathway [152].

### 4.5. Trigonelline (TGN)

In a study conducted by Kao CC et al., a unique genetic signature—the TGFβ3/GLI2/YAP1 (TGY) signature—was identified, which is significantly correlated with immune evasion and cisplatin resistance in BUC and plays a pivotal role in the transformation and functional regulation of CAFs, with TGFβ3 being particularly critical. *TGN* can form a stable complex with TGFβ3, which contributes to the downregulation of the TGY signature, resulting in a significant reduction in tumor spheroid formation ability, cisplatin resistance, and CAF transformation potential. *TGN* treatment also decreases the secretion of TGFβ3, IL-6, and VEGF by CAF-containing tumor-like constructs, all of which facilitate tumor-associated angiogenesis, drug resistance, and metastasis [153].

### 4.6. Triacanthine

*Triacanthine* effectively suppresses BUC growth and G1-S cell cycle transition both in vitro and in vivo, while inducing apoptosis and autophagy. It also upregulates early response kinases, including ERK and JNK, and induces caspase-dependent apoptosis and autophagy via the extrinsic pathway. Furthermore, *Triacanthine* mediates the suppression of migration and invasion in the human BUCs line EJ by inhibiting the binding of transcription factors: AP-1, Sp1, and NF-κB to MMP-9 [154].

**Figure 3:**
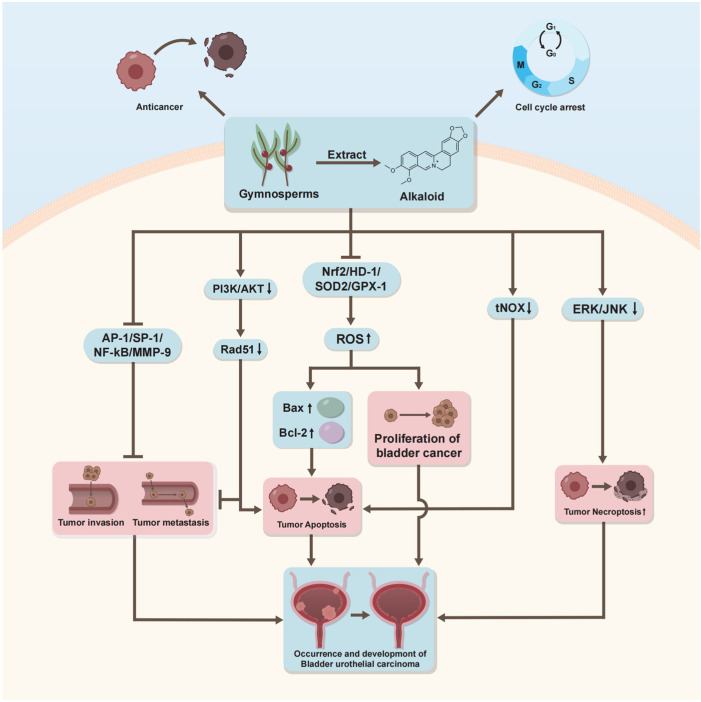
The Summary of the Mechanism by Which Alkaloids Exert Effects on Bladder Cancer Cells. *Triacanthine* suppresses cell migration and invasion while initiating extrinsic apoptosis. *Berberine* facilitates apoptosis through multiple pathways and initiates intrinsic apoptosis while also inhibiting cellular migration. *Capsaicin* can inhibit cell proliferation and induce apoptosis. Generated using Adobe Illustrator (Version 26.3).

MMP-9 is a calcium-dependent, zinc-containing protease that is closely linked to the migratory and invasive capacities of BUCs. Consequently, *Triacanthine* is considered to hold potential application value in suppressing the metastasis and progression of bladder tumors (Figure 3) (Table 7).

## 5. Other Natural Products

### 5.1. Anthraquinones

*Rhodopsin* (6-methyl-1,3,8-trihydroxyanthraquinone), a natural component found in different kinds of plants, including *Aloe vera* and *Polygonum multiflorum*, has gained much attention because it can stop inflammation, fight bacteria, and prevent tumors from growing [168]. Studies have shown that *Rhodopsin* has concentration-dependent inhibitory effects on the proliferation and invasion of T24 and 5637 BUCs [169]. Furthermore, the expression of Jagged1, VEGF, VEGFR2, and MMP-2 mRNAs and proteins is significantly reduced after *Rhodopsin* treatment, but overexpression of Notch1 can rescue the cell growth inhibition caused by *Rhodopsin*. Taken together, this implies that *Rhodopsin* inhibits the growth of BUCs via inhibition of the Notch1 pathway [169].

*Arbutin* (C12H16O7) is a phenylphenol glucoside, which can be found in many kinds of plants in nature, including Asteraceae and Rhododendronaceae, and it was discovered for the first time in the leaves of *Arbutus unedo* L. (Rhododendronaceae) [170]. The Transforming Cell Carcinoma of the Bladder Neck Interstitial Metaplasia human BUC line was used in a study, which demonstrated that *Arbutin* decreases the proliferation of this cell line in a dose- and time-dependent manner, and it is noteworthy that *Arbutin* does not have cytotoxic effects on this cell line when the concentration is below 500 mg/mL [171]. It is also known that *Arbutin* interferes with the cell cycle and deactivates ERK, and the interference of the cell cycle by *Arbutin* could possibly be due to the increase in cell cycle protein-dependent kinase inhibitor p21 (WAF1/C1P1) [171]. In summary, *Arbutin* inhibits the proliferation of BUCs through the inactivation of ERK and upregulation of p21.

### 5.2. Phenanthrenequinone

*Tanshinone IIA* (C19H18O3) (Tan-IIA) is a fat-soluble phenanthrenequinone compound extracted from the roots of the traditional Chinese medicine *Salvia miltiorrhiza* [172,173]. Tan-IIA has been found to inhibit the migration and invasion of BUCs [172]. Results indicate that it can inhibit the EMT, which is a crucial part of BUCs invasion and metastasis, through the inhibition of the STAT3 pathway and reduction in CCL2 expression in BUCs. Similarly, the rise in the epithelial marker E-calmodulin and the drop in mesenchymal markers N-calmodulin and waveform protein, along with the downregulation of transcription factors Snail and Slug, indicate that Tan-IIA treatment has the capability to prevent the EMT process [172].

### 5.3. Anthrone and Its Derivatives

*Garcinia acid* (GA) is a compound obtained from the natural resin of *Garcinia cambogia*, and it has been proven to have antiproliferative and pro-apoptotic effects on some types of tumors, such as breast cancer, prostate cancer, etc. [173,174]. GA is anticancer via many methods, such as causing cells to stop dividing, stopping telomerase, and stopping cells from invading [175,176]. It has been found that GA causes apoptosis in BUCs lines T24 and UMUC3 in a ROS-dependent manner [177].

**Figure 4:**
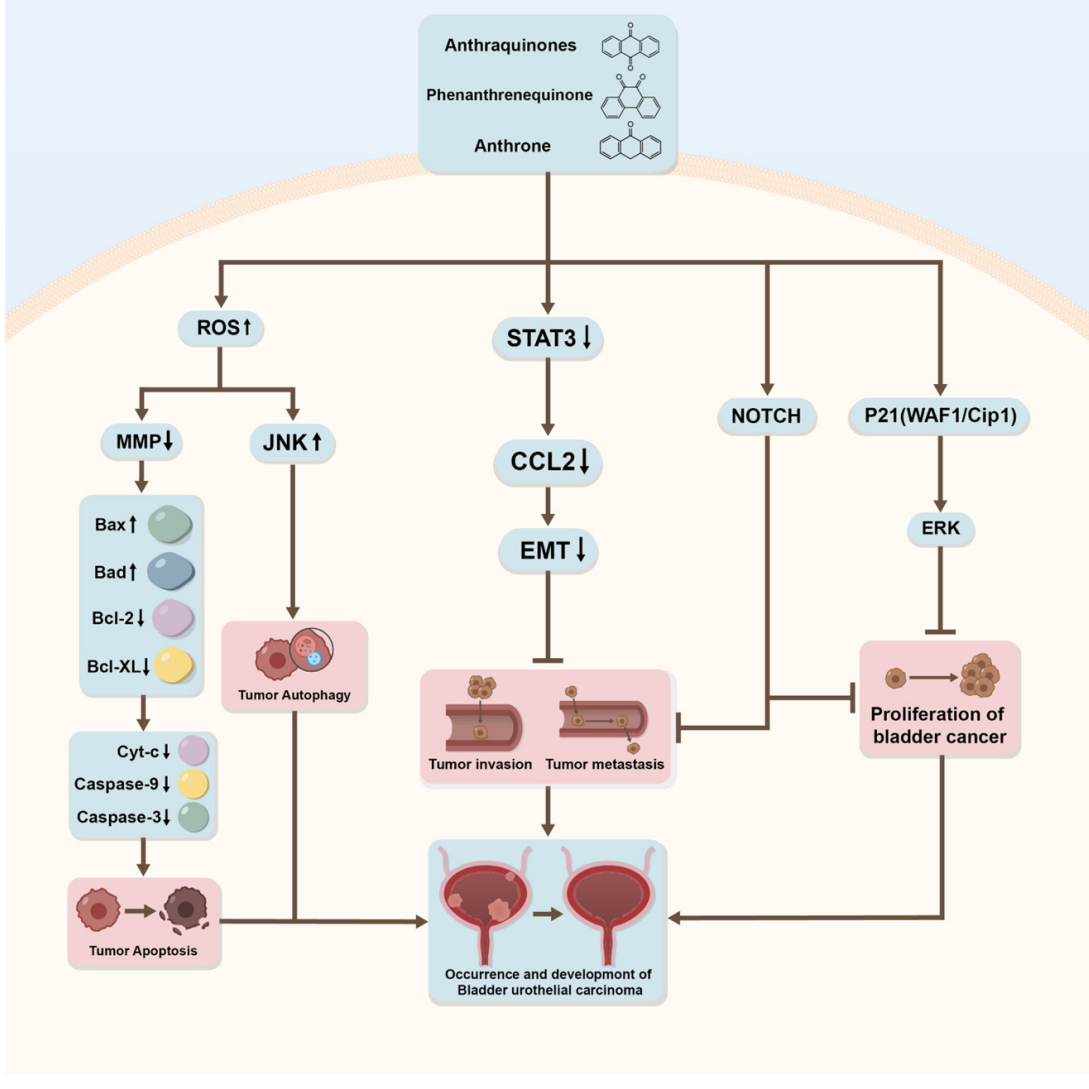
The summary of anticancer effects and mechanisms of *Rhodopsin*, *Arbutin*, TanshioneIIA, and Garcinia. *Rhodopsin* and *Arbutin* lead to the inhibition of cell proliferation and invasion. TanshioneIIA acts by inhibiting BCa’s invasion and metastasis. Garcinia may modulate signaling pathways, including intrinsic apoptosis and autophagy. Generated using Adobe Illustrator (Version 26.3).

GA generates ROS to activate the JNK pathway first, so there will be a big cell autophagy reaction, and then it stops later when the caspases are activated. Then, GA causes ROS-mediated caspase activation, which leads to the breakdown of different Atg proteins, as well as mitochondrial polarization and cytochrome c release, thus activating the intrinsic apoptotic pathway. Also noteworthy is that GA-induced autophagy is a cell survival response that postpones caspase activation. Moreover, this research also indicates that the inhibition of NF-κB by GA is realized via the inhibition of IκB-α, rather than through the degradation of Hsp90 or IKK, which happens prior to caspase activation [177] (Figure 4) (Table 8).

## 6. Discussion

### 6.1. The Main Therapeutic Effects of Natural Products in the Treatment of Bladder Cancer

In existing research on natural products in the field of BUC treatment, the primary focus has been on inducing apoptosis or pyroptosis in BUCs [25,32,53,72,79,114,131,138,143,152,177], inhibiting cell proliferation through cell cycle arrest [34,80,130,131,145,173,174] and angiogenesis inhibition [53,84,153], suppressing cell invasion and migration [24,44,66,72,82,98,121,125,132,143,150,172], enhancing the therapeutic effects of conventional drugs [45,65,108,112], inhibiting drug in BUCs, suppressing immune escape [67,75,84,85,153], and remodeling the tumor microenvironment [67,111,114].

It is also important to note that no matter what kind of natural product it is, its regulation of bladder cancer survival and metabolism mainly occurs by regulating the MAPK family signal transduction pathway [42,62,63,78,94,95,96,97,113,121] and the PI3K/Akt/mTOR pathway [26,36,99,100,126]. The downstream effects of these signaling pathways include the biological activities of the above-mentioned natural products. Also, the most common results in this study are about different ways that have Bax, Bcl-xL, caspase-9, and caspase-8 proteins [25,32,53,72,79,114]. Caspase-dependent apoptosis is connected to the downstream consequences of the MAPK and PI3K/Akt/mTOR signaling pathways; however, most of the work in this area has only reached the identification of downstream pathways. Therefore, creating a more complete regulatory signaling pathway connecting these two parts should be a major point for later research.

In terms of immune evasion suppression [67,153] in bladder cancer cells and the regulation of the tumor microenvironment [67,84,111,114], most of the research work has been done from the material point of view, studying and analyzing the possible application of nanomaterials in this field. And it involves studies about some natural things changing how many certain types of immune cells called T cells are in tumors. But the number of papers on this topic is not as many as the ones about the signal pathways that were talked about before. Thus, this viewpoint should also be developed and considered.

### 6.2. The Potential Synergistic Effects Between Natural Products and Chemotherapeutic Agents in the Treatment of Bladder Cancer

Regarding the potential synergistic effects between natural products and conventional chemotherapeutic agents, the primary mechanisms can be categorized as follows: Improving the cytotoxicity of traditional chemotherapeutic drugs [45,65,108], overcoming or reversing chemotherapy resistance, reducing the organ toxicity caused by traditional drugs, and regulating the tumor microenvironment (TME) and immune response. Conventional chemotherapeutic drugs such as vincristine, cisplatin, BCCG [27,130].

In general, natural products form a cooperative network to improve traditional chemotherapy, reverse resistance [63,64,101,105,107,108,153], and regulate immunity. By intervening in key signaling pathways such as PI3K/AKT/mTOR, MAPK, STAT3, and Wnt/β-catenin, as well as interacting with various forms of cell death, including apoptosis, autophagy, and ferroptosis. But there are still many knowledge gaps in existing research. Take monoterpenes and tetraterpenes, for example, the mechanism of action is not clear, and lack of basic research limits their clinical application. Therefore, it is necessary to deepen the study of these synergistic mechanisms in the future and promote the development of basic and translational research of these relatively unexplored natural products, which will be important for the development of this field.

### 6.3. Limitations and Obstacles for Clinical Translation

Among them, *paclitaxel* from the diterpenoid class has gone through several clinical trials and has demonstrated considerable effectiveness [54,55,56,57,58,59,60,61], making it an ideal substitute therapy for BUC patients who have developed resistance to drugs.

In the exploration of treatments for BUC, the clinical translation of natural products has demonstrated remarkable progress. As a representative of diterpenoid compounds, *paclitaxel* has been proven through a series of clinical studies to possess significant therapeutic efficacy, providing an excellent alternative treatment option for patients with drug resistance. This case highlights the crucial value of natural products, exemplified by taxane drugs, in bladder cancer treatment. Current studies focus on mixing them with other medicines that work differently to make new ways to treat patients better.

For example, the combination regimen of *paclitaxel* and albumin-bound *paclitaxel* with immune checkpoint inhibitors (such as Socazolimab, Tislelizumab, Avelumab) or targeted drugs (such as trastuzumab analogs, everolimus) in the neoadjuvant or first-line treatment of advanced urothelial carcinoma and MIBC showed a good synergistic effect and clinical activity [54,56,61], especially providing an effective option for cisplatin-unfit patients. Also, *paclitaxel* paired with radiation was feasible for keeping organs intact [57]. But it is obvious that the current clinical trials are mainly at Phase I or II [54,55,56,57,58,59,60,61]. However, to bring a drug to clinical application, it is necessary to determine its safety in Phase I and screen for indications in Phase II before entering Phase III, which is the most costly and challenging stage of research and development to confirm the efficacy of the drug. Therefore, there is still a long way to go for the relevant clinical trials in this field.

Clinical explorations have been driving key translational research directions such as identifying biomarkers for combination therapy (PD-L1 expression, tumor mutational burden [61]), and explaining how they work together [53]. Clinical trial designs that include both cisplatin-sensitive and -resistant groups (for example, Oncodistinct 004—AURA trial) [178] are moving towards personalized treatments. But still, big random studies need to prove if the combo mix works over a long time and help put new things, like special antibodies mixed with medicine, into the best ways to treat people.

### 6.4. The Limitations of the Use of Natural Products in the Management of Bladder Urothelial Carcinoma

Although the results from the related studies show that there are certain pathways and mechanisms of action for anticancer therapy, these studies were mainly done with cells and animals, so we still don’t know if these can be applied to clinical treatments. Therefore, more clinical trials should be carried out, similar to the ones for *paclitaxel*, but before doing so, it is necessary to establish the drug concentration for the study because some natural products have markedly different effects at different concentrations [34,43,44,130,171]. Moreover, even in the case of cell and animal experiments, some natural products also show low bioavailability and poor stability, such as *Astragaloside IV* and *curcumin*. But related studies have already pointed out the direction of research to solve the problem of low bioavailability, that is, using a nanodelivery system to achieve accurate therapeutic effect [84,109,110].

## 7. Conclusions

To sum it up, for future research on natural products in BUC treatment, we need to improve clinical studies and carry out animal and cell experiments in some unclear areas, such as monoterpenes, tetraterpenes, etc. Additionally, some natural products may require additional purification and separation processes to produce less toxic and more bioavailable products, which will also be an important direction for drug development in this area [179,180].

## Figures and Tables

**Table 1:** The summary of the roles of Monoterpenes in combating BUC.

Terpene Classification	Chemical Compound	Characteristic	Efficacy	Research Type	Role in BUC	Mechanism
Monoterpenes	*EO-ACs*	Complex natural compounds exhibit a wide range of functions in regulating biological activities, making them valuable therapeutic agents for treating various diseases and conditions. Most of these are terpenoid compounds.	Anti-tumor, anti-inflammatory, anti-oxidant	in vitro	Induction of endogenous apoptosis in BUCs [24]	*EO-Acs* induces intracellular ROS production, leading to a decrease in ΔΨm and inducing mitochondria-dependent endogenous apoptotic pathways
					Antimetastatic [24]	Reduces MMP-9 levels to some extent
	*limonene*	A monocyclic monoterpene hydrocarbon that exists in the form of three cyclic isomers called d-*limonene*, l-*limonene*, and DL-*limonene*	Protects against free radicals and lipid peroxidation	in vitro	Induction of apoptosis and inhibition of proliferation in BUCs [26]	1. Combination with cisplatin results in inhibition of the PI3K/AKT/mTOR signaling pathway, inhibiting the proliferation of BUCs 2. The inhibition of AKT phosphorylation induced the onset of apoptosis
	*Genipin* (GP)	A terpenoid compound widely used in the pharmaceutical industry as a cross-linker and drug delivery agent in the synthesis of various biopolymers. Due to its natural solubility and low cytotoxicity, GP has been used in the development of novel cross-linking agents. In addition, GP is a backbone for the synthesis of various alkaloids in synthetic medicinal chemistry.	It can improve liver ischemia–reperfusion injury, steatosis, autoimmune hepatitis, and fibrosis in rodents. It can also inhibit hyperglycemia-induced renal tissue damage by reducing oxidative stress and inflammation	in vivo	Reducing renal injury during cisplatin treatment of BUC [27]	1. Oxidative stress was counteracted by attenuating the cisplatin-induced increase in the activity of NADPH oxidase and by reducing the cisplatin-induced accumulation of ONOO-, a potent oxidant associated with nitrative stress in renal tissues, to counteract oxidative stress and nitrative stress, respectively. 2. Inhibition of cisplatin-induced NF-κB activation and expression of pro-inflammatory cytokines (TNF-α and IL-1β) to counteract inflammation. 3. Inhibition of cisplatin-induced MAPK activation counteracts cisplatin-induced inhibition of renal cell proliferation. 4. Reduction in caspase 3 and PARP activity in cisplatin-treated renal cells and protection of DNA from damage to counteract apoptosis

**Table 2:** The summary of the roles of Sesquiterpenes in combating BUC.

Terpene Classification	Chemical Compound	Characteristic	Efficacy	Research Type	Role in BUC	Mechanism
Sesquiterpene	*Artesunate* (ART)	Water-soluble derivatives of artemisinin, of which *Artesunate* derivatives are the most rapid-acting and effective of all antimalarials [30]	In addition to its well-known antimalarial effects, *Artesunate* has now been analyzed for its anticancer activity in 55 cell lines from the Developmental Therapeutics Program of the US National Cancer Institute [31].	In vivo [32]	Induction of apoptosis in BUCs	1. By increasing the expression of miR-16 protein in T24 cells, the downstream expression of COX-2 is inhibited, leading to a reduction in PGE2 protein expression, thereby promoting tumor cell apoptosis [32]. 2. By upregulating ROS levels, the AMPL-mTOR-ULK1 pathway is activated, thereby inhibiting the synthesis of Bcl-2 protein downstream and promoting the synthesis of cleaved-caspase3 and cleaved-PARP proteins, which induce caspase-dependent apoptosis, and this apoptosis is autophagy-induced [33].
				In vitro [33]		
				In vitro [34]	Induction of cell cycle arrest in cisplatin-resistant BUCs	Triggering G0/G1 cell cycle arrest by blocking G1/S transition in cisplatin-resistant BUCs [34]
					Induction of autophagy-dependent apoptosis in cisplatin-resistant BUCs	At higher concentrations, ART (10 μM) triggered mitochondrial dysfunction and activated autophagy, which mediated autophagy-dependent apoptosis in cisplatin-sensitive and cisplatin-resistant BUCs [34]
				In vivo [35]	*Artesunate* -cisplatin treatment pretreatment attenuates cisplatin-induced renal damage.	The antimalarial drug *Artesunate*, following pretreatment with cisplatin at a therapeutic dose, not only enhanced the efficacy of high-grade uroepithelial carcinomas in male albino mice through inverse gene expression modulation of FGFR3, HRAS, P53, and KDM6A but also significantly reduced cisplatin-induced renal injury [35]
	*Atractylenolide* (ATR)	Sesquiterpenoids from the traditional Chinese medicine *Atractylodes macrocephala* Koidz [36]	Anti-cancer, anti-inflammatory, anti-platelet, anti-osteoporosis, and anti-bacterial activity; protects the nervous system; and regulates blood sugar and blood lipids [36]	In vivo And In vitro	Induction of apoptosis in BUCs	1. Triggers apoptosis through activation of the mitochondrial apoptotic pathway 2. Depends on inhibition of the PI3K/Akt/mTOR signaling pathway, leading to a further reduction in the up-regulation of apoptosis-related proteins Bax and Bad [37]
					Induction of cell cycle arrest in BUCs	ATR upregulates p21 and downregulates cyclin B1, CDK1, and Cdc25c, causing cell cycle arrest in G2/M phase [37].
					Inhibition of tumor cell growth in mice	ATR-1 blocks T-24 or 253J-induced xenograft tumor growth and is well tolerated by the body [37]
	*Scabertopin scaber* L.	Scabertopin is one of the major sesquiterpene lactones found in *Elephantopus scaber* L. [43]	*Scabertopin scaber* L. is thought to have considerable anticancer properties [43].	In vitro	Induction of necrotic apoptosis in BUCs	Activates RIP1/RIP3/MLKL phosphorylation by lowering ΔΨm and stimulating mitochondrial ROS production, thereby mediating necrotic apoptosis in J82 cells [43]
					Inhibition of BUCs migration	Inhibition of FAK/PI3K/Akt signaling pathway inhibits the expression of MMP-9, which ultimately inhibits the migration and invasion of BUCs [43].
	*Antrocin*	A medicinal mushroom, widely used for centuries in Asia and Europe for immunomodulation, cancer prevention, and liver protection.	Antibacterial, antioxidant, and anti-tumor properties	In vitro	Inhibition of BUCs migration and invasion at non-cytotoxic concentrations	Inhibition of migration and invasion of human BUCs through inhibition of ERK/FAK/pilein and ERK/c-Fos/MMP-2 signaling pathways at non-cytotoxic concentrations [44].
					Induction of exogenous and endogenous apoptosis in BUCs at cytotoxic concentrations	At cytotoxic concentrations leads to elevated expression of proteins such as Fas, DR5, and Bax, which mediate the onset of exogenous and endogenous apoptosis, respectively [44].
	*cis-Nerolidol*	An aliphatic sesquiterpene alcohol with an asymmetric centre at the C-3 position and a double bond at the 6position, allowing it to exist in four stereoisomeric forms. It occurs naturally in the *essential oils* of various plants, has a floral aroma, and is usually a mixture of all four isomers.	Nerol has antioxidant, anti-injurious, antimicrobial, antiparasitic, and antitumor activities.	In vitro	Inducing cell death in BUCs	1. In the early stage of treatment, it can signal through β-adrenergic receptors, PKA, and sAC to increase the calcium transport activity of RYR in the endoplasmic reticulum to the cytoplasm, and the increase in intracellular calcium induces endoplasmic reticulum (ER) stress, downstream activation of the p38-MAPK pathway, and caspase-1-dependent cell death [41]. 2. In the late stage of treatment, prolonged high Ca^2+^ concentration in the cytoplasm further induces the production of ROS, which leads to prolonged activation of ERK downstream of it, which in turn induces paraptosis [41].
					Inhibition of cell cycle arrest in BUCs	G1 phase accumulation of BUCs occurred after cis-Nerolidol treatment, producing an inhibitory effect on BUCs proliferation [41].
	*(-)-Gochnatiolide B*	*(-)-Gochnatiolide B* is a guaiacolide-type sesquiterpenoid dimer first isolated from the roots of *Gochnatia pomculat* [40]	Bioactivity studies have shown that *(-)-Gochnatiolide B* significantly inhibits the growth of a variety of cancer cells, including liver, lung, ovarian, colon, and prostate cancers, especially BUC.	In vivo And In vitro	Induction of apoptosis in BUCs	Increased protein levels of p21 and p27 lead to G1-phase blockade, which in turn induces caspase-dependent apoptosis [40].
	*Mogoltacin*	*Mogoltacin* is a sesquiterpene coumarin from *Ferula badrakema*. *Mogoltacin* was isolated from the fruit of *F. badrakema* using silica gel column chromatography and preparative thin-layer chromatography [45].	These sesquiterpene coumarins have a drimane-type structure. There is some evidence that these compounds can enhance the cytotoxicity of chemotherapeutic drugs by inhibiting P-glycoprotein (P-gp).	In vitro	Enhancement of Vincristine Cytotoxicity in Human TCC Cell Lines	Enhances vincristine cytotoxicity in human TCC cell lines by competitively blocking P-gp transporter [45]
	*Parthenolide*	*Parthenolide* (PTL) is a sesquiterpene lactone isolated from Tanacetum parthenium, an herb traditionally used for the relief of migraine, inflammation, and rheumatoid arthritis.	Has been shown to exhibit anticancer activity by inducing apoptosis and necrosis in a variety of solid tumors	In vitro	Induction of cell cycle arrest in BUCs	Causes G1 phase cell cycle arrest in 5637 cells by regulating cell cycle protein D1 and phosphorylating cell cycle protein-dependent kinase 2 [46].

**Table 3:** The summary of the roles of Diterpenes in combating BUC.

Terpene Classification	Chemical Compound	Characteristic	Efficacy	Research Type	Role in BUC	Mechanism
Diterpenes	*paclitaxel*	A tetracyclic diterpenoid, first isolated from the bark of the Pacific yew tree, *Taxus baccata* [49]	At present, *paclitaxel*, as a low-toxicity, high-efficiency, and broad-spectrum natural anticancer drug, has been widely used in the treatment of ovarian, breast, and uterine cancers [50].	In vivo [51]	In combination with anti-epidermal growth factor receptor monoclonal antibody C225 [51]	1. C225 can downregulate Raf-1 kinase activity, thereby enhancing *paclitaxel*-induced cytotoxicity. 2. *Paclitaxel*, in turn, can increase C225’s anti-angiogenic and anti-metastatic capabilities by downregulating the expression of basic fibroblast growth factor, vascular endothelial growth factor, interleukin-8, and matrix metalloproteinase-9.
				In vivo [52]	Conjugated to an anti-vascular endothelial growth factor receptor monoclonal antibody DC101 [52]	Demonstrate a high rate of apoptosis of vascular endothelial cells and tumor cells.
				In vitro and In vivo [53]	Treatment of metastatic human metastatic cell carcinoma with the angiogenesis inhibitor TNP-470 (AGM-1470) [53]	Effects are at least partially mediated by inhibition of angiogenesis, expression of bFGF and MMP-9, and induction of apoptosis.
				clinical trials	A series of clinical trials in combination with other chemotherapeutic agents	Including studies on extremely high-risk non-muscle-invasive BUC [54], preoperative muscle-invasive BUC patients [55], HER2-positive recurrent or metastatic urothelial carcinoma [56], muscle-invasive BUC in non-cystectomy candidates [57], post-operative muscle-invasive BUC [58], BCG-unresponsive non-muscle-invasive bladder urothelial carcinoma [59], cisplatin therapy for advanced urothelial carcinoma [60], and unspecified advanced urothelial carcinoma [61]. All showed good drug tolerance and lower adverse reactivity than monotherapy.
	*Jolkinolide B*	A natural bicyclic diterpenoid from the traditional Chinese medicine *Euphorbia fischeriana* Steud.	Increasing interest due to its profound anti-tumor activity	In vitro [62];	Induction of cell death in BUCs [62]	1. By targeting the thioredoxin and glutathione systems in BUCs, TrxR1 inhibition and GSH depletion induced ROS production, while excessive ROS up-regulated the expression of p-JNK, p-p38, and p-ERK in T24 and UM-UC-3 cells, which activated the MAPK/ERK pathway and induced paraptosis in the tumor cells. 2. At the same time, excessive ROS production also induces ER-stress, which further aggravates paraptosis; 3. In addition to paraptosis, ROS-induced overexpression of p-JNK, p-p38, and p-ERK induces apoptosis.
				In vitro and in vivo [63]	Enhanced sensitivity of BUCs to mTOR inhibitors [63]	Previous studies have demonstrated that the use of mTOR inhibitors leads to the activation of Akt feedback and the development of cytoprotective autophagy, whereas *Jolkinolide B* can sensitize BUC to mTOR inhibitors through dual inhibition of Akt signaling and autophagy.
				In vitro and in vivo [64]	Synergistic enhancement of the antitumor activity of GPX4 inhibitors [64]	Synergistic enhancement of the antitumor activity of GPX4 inhibitors by inhibiting TrxR1 in cisplatin-resistant BUCs and further mediating iron death and apoptosis in cisplatin-resistant BUCs through the induction of lipid reactive oxygen species.
	*Abietic acid* (AA)	A naturally occurring diterpene compound from *Pinus palustris* and *Pimenta racemose var. grissea*	Possesses different pharmacological activities, including anti-inflammatory, anticonvulsant, anti-obesity, anti-allergic, and anti-tumor activities	In vitro and in vivo [65]	Induction of iron death in BUCs [65]	By activating the HO-1 pathway in BUCs, GPX4 was subsequently inhibited, so the use of AA induced ferroptosis in BUCs.
					Synergistic enhancement of the efficacy of conventional chemotherapeutic agents [65]	AA significantly increased cell death induced by cisplatin, *paclitaxel*, gemcitabine, and gefitinib, all of which exhibited synergistic effects.
	New aphidicolin diterpenoids from the deep-sea-derived fungus *Botryotinia fuckeliana*	Since the discovery of the first aphidicolin-type diterpenoid, aphidicolin, from *Cephalosporium aphidicola Petch*, about 100 naturally occurring aphidicolin species have been reported, and the aphidiconlin in the present experiments is derived from the deep-sea source fungi *Botryotinia fuckeliana*	Possesses significant antiviral, cytotoxic, and root growth inhibitory activities as well as antitumor potential. cytotoxicity and root growth inhibitory activity, as well as anti-tumor potential	In vitro [66]	Inhibition of cell cycle arrest in BUCs [66]	Anti-T24 cells by reducing the protein levels of CDK4, CDK6, and cyclin D1 in T24 cells, thereby blocking T24 cells in G0/G1 phase.
					Inhibition of BUCs migration [66]	Inhibition of T24 cell migration was demonstrated by transwell assays.
	*Oridonin*	a bioactive diterpenoid component isolated from *Rabdosia rubescens* (Hemsl.) [68]	Possesses considerable anticancer activities, such as induction of cell cycle arrest, cell death due to apoptosis, and inhibition of angiogenesis	In vitro and in vivo [67]	Regulation of BUC metabolism [67]	Inhibits Warburg effect-mediated BUC survival and immune escape by covalently targeting and binding to HK1 and thereby inhibiting glycolysis in BUCs.
					Modulation of the tumor immune microenvironment (TME) [67]	*Oridonin* combined with PD-L1i enhances the antitumor effect of the tumor immune microenvironment by alleviating immunosuppression and increasing the amount of cytokines secreted by CD8+ T cells.
	*Ovatodiolide* (OV)	A macrocyclic bioactive component isolated and purified from *A. indica*	It has been confirmed to have anti-inflammatory properties.	In vitro and in vivo [69]	Modulation of the tumor immune microenvironment (TME) [69]	Bladder carcinogenesis can be inhibited by suppressing mTOR/β-catenin/CDK6 and exosomal miR-21 release from M2 tumor-associated macrophages.

**Table 4:** The summary of the roles of Triterpenoid in combating BUC.

Terpene Classification	Chemical Compound	Characteristic	Efficacy	Research Type	Role in BUC	Mechanism
Triterpenoid	*Betulinic acid*	A pentacyclic triterpenoid in the bark of *Betula* sp. [71]	BA has been reported to possess a wide range of biological activities, including antibacterial, antiviral, anti-inflammatory, antioxidant, antithrombotic, antifibrotic, hepatoprotective, antiangiogenic, antitumor effects	In vitro [72] In vitro [73]	Induction of apoptosis in BUCs	1. It can cause a decrease in intracellular Bcl-2 expression and an increase in Bax content in BUC lines, further inducing the activation of the endogenous mitochondria-dependent apoptotic pathway. 2. Experiments further demonstrated that *Betulinic acid* further induces apoptosis in human BUCs induced partially due to the arrest of the G2/M phase by down-regulating the expression of G2/M-associated proteins [72].
						Induction of autophagy-dependent apoptosis via the Bmi-1/ROS/AMPK-mTOR-ULK1 axis [73].
					Inhibition of BUCs migration	The decrease in MMP-9 levels in various BUC lines after treatment with *Betulinic acid* has been demonstrated [72].
	*Maslinic acid*	A naturally occurring triterpenoid found in abundance in olives and widely distributed in a variety of foods.	Antioxidants, anti-inflammatory agents, antiviral agents, antimalarials, antiprotozoal agents, and antidiabetic agents	In vitro [78]	Induction of apoptosis in BUCs	1. They induced apoptosis in BUCs by activating the p38 MAPK signaling pathway 2. They also demonstrated that *Maslinic acid* was selective in this apoptosis-inducing effect in animal studies [78].
	*Pachymic acid*	A lanostane-type triterpenoid derived from *Poria cocos mushroom*	Has a variety of beneficial properties, such as anti-cancer, anti-inflammatory, anti-oxidant, and anti-metastasis [79]	In vitro [79]	Induction of apoptosis in BUCs	Induction of caspase-dependent apoptosis by an exogenous pathway through upregulation of TRAIL receptors and further induction of related endogenous apoptotic pathways through modulation of Bcl-2 and IAP family members in EJ BUCs [79].
	β-escin	An active pentacyclic triterpene, derived from the seeds of *horse chestnuts*	Exhibits a variety of biological activities including anticancer, anti-edema, anti-inflammatory, antioxidant, and vasorelaxant activities.	In vitro and in vivo [86]	Induction of apoptosis in BUCs	1. Induces caspase-dependent apoptosis through up-regulation of reactive oxygen species production and loss of mitochondrial membrane potential 2. Induces apoptosis through the exon pathway (death receptor), which involves the activation of the Fas (CD95) death receptor/FADD as well as caspase-8 and caspase-3 [86]
					Inhibition of BUCs proliferation	Inhibits the growth of BUCs by inhibiting angiogenesis [86].
	*Asiaticoside* (AC)	A natural triterpene derivative from *Centella asiatica*	Possesses various biological activities, such as anti-inflammatory, wound healing, osteoclastogenesis, anti-fatigue activity, antioxidant activity, anti-fibrotic effect, and neuroprotection	In vitro and in vivo [75]	Induction of iron death in BUCs	Combination with propofol, a local anesthetic drug with antitumor activity, resulted in increased intracellular Fe^2+^ expansion and ROS levels in BUCs and decreased expression of xCT and GPX4, inducing iron death [75].
					Inhibition of BUCs migration	Also, in combination with propofol, the levels of decreased N-calmodulin level content and increased E-calmodulin level content in BUCs were more significant, suggesting that the EMT process in BUC was inhibited [75]
					Inhibition of the immune escape of BUCs	1. The percentage of CD8+ T cells and the magnitude of the increase in IFN-γ concentration 2. The decrease in the level of PD-L1 expression in BUCs was also increased after the same combination, suggesting that the probability of immune escape was reduced [75].
	*Brusatol*	A bitterwood analogue 1 obtained from *Brucea javanica*, an evergreen shrub growing in Southeast Asia, and a highly oxidized class of triterpenoid derivatives endemic to plants of the *Simaroubaceae*.	Ability to induce a range of biological responses and exert anti-inflammatory, anti-leukemia, and anti-cancer activities	In vitro and in vivo [81]	Induction of iron death in BUCs	*Brusatol* induces iron death in BUCs by blocking the Chac1/Nrf2/SLC7A11 pathway, leading to a disruption of redox homeostasis: a decrease in the level of GPX4 and an accumulation of intracellular ROS and Fe^2+^ [81].
	*Frondoside A*	*Frondoside A* is a triterpene glycoside isolated and purified from *Cucumaria frondosa*.	It is known to have antiproliferative, anti-invasive, anti-angiogenic, anticancer, and immunomodulatory effects.	In vitro and in vivo [82]	Induction of apoptosis in BUCs	The apoptosis is not dependent on caspases or TP53, and the exact mechanism is not well understood [82].
	*β-amyrin*	A widely distributed plant in the Amazon and in the Northeast of Brazil, commonly known as *almécega*	Previous studies have described its antimicrobial, anti-inflammatory, gastroprotective, and anti-injury properties.	In vitro [80]	Triggering cell cycle arrest-induced apoptosis.	The accumulation of ROS in NTUB1 cells after treatment induces cell cycle arrest and triggers apoptosis [80].
	*Platycodin D* (PD)	The major saponins extracted from Platycodonis radix	PD exerts antitumor effects in a variety of cancers, such as lung cancer, gastric cancer, hepatocellular carcinoma, and so on.	In vitro and in vivo [83]	Induction of apoptosis in BUCs	Down-regulates LncRNA-XIST and promotes miR335 expression, which inhibits the generation of malignant phenotypes in BUC and promotes apoptosis in BUCs in a dose- and time-dependent manner [83].
	*Astragaloside IV*	*Astragaloside IV* (AS-IV) is a glycoside of the cyclic spinach triterpenoids, a widely used Chinese herbal medicine derived from *Astragali radix*. Has been widely used in Chinese medicine to treat wounds, anaemia, and chronic fatigue [adding]	Possesses a wide range of biological activities, including promotion of angiogenesis, amelioration of ischemia/reperfusion injury, and regulation of glucose and lipid metabolism	In vitro and in vivo [84]	Inhibited tumor angiogenesis	*Astragaloside IV* exerts a potent dual inhibitory effect on STAT3 and NF-κB in MB-49 tumor cells, thereby inhibiting tumor angiogenesis [84].
					Synergistic aPD-L1 regulation of the tumor microenvironment	Synergistic aPD-L1 promoted CD8+ T cell infiltration and activation and reduced regulatory T cell (Treg) infiltration in tumors [84].

**Table 5:** The summary of the roles of Tetrapene in combating BUC.

Terpene Classification	Chemical Compound	Characteristic	Efficacy	Research Type	Role in BUC	Mechanism
Tetrapene	*Fucoxanthin*	A Marine-Derived Carotenoid	Possesses a wide range of pharmacological activities, including antioxidant, anti-inflammatory, anti-obesity, and anticancer effects	In vitro [89]	Induced apoptosis in BUCs	It can inhibit the expression of STAT3 in TCC cell lines, which in turn downregulates the expression of BCL-xL, which in turn leads to a decrease in the effect of inhibiting apoptosis in BUCs and promotes apoptosis [89].
					Increased sensitivity of BUCs to cisplatin	Increased sensitivity of TCC cell lines to cisplatin, as demonstrated by a series of in vitro studies [89].

**Table 6:** The summary of the different types of phenols’ functions in combating BUC.

Phenols	Source and Nature	Efficacy	Effects on BUC	Molecular Mechanisms	Research Type
*Curcumin*	Orange-yellow natural compound with anti-inflammatory and antioxidant properties	Anti-tumor, anti-inflammatory, anti-oxidant, enhances chemosensitivity	Anti-tumor effect by blocking proliferation and inducing apoptosis of cancer cells	1. Inhibits Trop2/Cyclin E1 and upregulates p27 [98].	In vitro
				2. Block IGF-2/IGF-1R and HER-2 signaling, inhibit PI3K/AKT/mTOR/MAPK pathway [99,100].	In vitro
				3. Inhibits YAP/TAZ expression, promotes ubiquitin-proteasome degradation of the pro-oncogenic transcription factor KLF5, and downregulates Cyclin D1 [101].	In vitro
			Anti-metastatic	1. Reverses EMT through ERK5/AP-1 pathway and eliminates cancer stem cell properties [103].	In vitro
				2. Reverses EMT through Wnt/β-catenin pathway and eliminates cancer stem cell properties [104].	In vitro and in vivo
			Overcoming drug resistance	1. Inhibit NF-κB, up-regulate TRAIL, and enhance BCG efficacy [106].	In vitro and in vivo
				2. *Curcumin*–dichloroacetic acid hybrid molecules target multidrug-resistant cells [107].	In vitro and in vivo
				3. Inhibits Aurora A kinase and induces autophagy to selectively kill BUCs and potentially bypass DNA repair/drug efflux [108].	In vitro
*Apigenin*	Natural flavonoid with multidimensional inhibitory effects	Anti-inflammatory, antioxidant, anticancer; regulates tumor microenvironment	Inhibits proliferation, metastasis, and induces apoptosis	1. Blocked BUC T-24 cells in G2/M phase by regulating cell cycle-related proteins, such as reducing cyclin B1 and CDK1 expression, and this cycle blocking effect was positively correlated with the dose [112].	In vitro
				2. Up-regulation of Bax, down-regulation of Bcl-2, and activation of the caspase cascade reaction [112].	In vitro
				3. Inhibits uPAR expression, blocks MAPK pathway phosphorylation, and blocks AP-1 and NF-κB activity [113].	In vitro
*Luteolin*	Natural flavonoids in fruits and vegetables, both antioxidant and pro-oxidant properties	Anti-inflammatory, anti-cancer, multifunctional therapy	Inhibit tumor growth	1. Inhibits mTOR/S6K phosphorylation and blocks protein synthesis [116].	In vitro and in vivo
*Baicalein*	Derived from Scutellaria baicalensis, broad-spectrum antitumor activity	Inhibits tumor proliferation, invasion, and induces apoptosis	Inhibits proliferation, invasion, and induces apoptosis	1. Activates exogenous/endogenous apoptotic pathway, ROS-dependent triggering of mitochondrial disruption, up-regulates Bax and down-regulates Bcl-2, and activates caspase-8/-9/-3 cascade response [119].	In vitro
*Scutellarin*	*Scutellaria baicalensis* extract, low toxicity, strong targeting properties	Anti-tumor and anti-metastasis; protects normal cells	Inhibits migration, invasion, and metastasis in vivo	1. Inhibits PI3K/Akt and MAPK pathways, blocks hypoxia-induced EMT [121].	In vitro and in vivo
*Tangeretin*	Natural flavonoids target mitochondrial function, with low cytotoxicity properties.	Antioxidant and pro-oxidant; targets energy Metabolism	Inhibits proliferation and induces apoptosis	1. Decreases mitochondrial membrane potential, increases cytochrome C release, modulates Bax/Bcl-2 ratio, and activates caspase-9/-3 [122].	In vitro
				2. Inhibits oxidative phosphorylation and increases ROS production [122].	In vitro
EGCG	Natural flavanol polyphenol, widely found in *Camellia sinensis*	Antioxidant, anti-inflammatory, modulates epigenetics; reverses chemoresistance, inhibits relapse	Inhibits proliferation and migration; targets cancer stem cells; enhances chemosensitivity	1. Down-regulates NF-κB/MMP-9; inhibits proliferation and migration of BUC SW780 cells [125].	In vitro
				2. Inhibits the PI3K/AKT/mTOR pathway and induces autophagy-dependent apoptosis [126].	In vitro
				3. Inhibits the SHH pathway and blocks stem cell self-renewal [127].	In vitro
*Isoliquiritigenin*	Licorice-derived chalcone, low nephrotoxicity	Synergizes chemotherapeutic potentiation and antagonizes cisplatin nephrotoxicity	Reduces cancer cell viability; enhances cisplatin efficacy and protects the kidney	1. Reduces viability of BUC T24 cell line at 15 μM and 25 μM concentrations; antagonizes cisplatin nephrotoxicity; regulates antioxidant proteins to protect kidney cells [130].	In vitro
*Licochalcone A*	Licorice active chalcone, pro-oxidant properties	Induces DNA damage and triggers apoptosis	Inhibits proliferation and induces apoptosis	1. Increases ROS accumulation, causing DNA damage [131].	In vitro
				2. Causes cell cycle arrest in G2/M phase and activates mitochondria-dependent apoptosis [131].	In vitro
*Echinatin*	Multi-targeted licorice chalcone, low normal cytotoxicity	Synergizes chemotherapy to inhibit metastasis and reverse EMT	Inhibits proliferation, migration, and invasion; enhances chemosensitivity	1. Down-regulates cyclin B1 and CDK1 expression, induces G2/M-phase cell cycle arrest, while inhibiting the activity of proliferation markers PCNA and c-Myc [132].	In vitro and in vivo
				2. Activates p38 phosphorylation to promote apoptosis [132].	In vitro and in vivo
				3. Enhances phosphorylation of GSK3β-Tyr216 site and inhibits Wnt/β-catenin pathway [132].	In vitro and in vivo
				4. Reverses EMT and synergizes with cisplatin/gemcitabine [132].	In vitro and in vivo
*Genistein*	Soy-derived isoflavone with significant dietary relevance	Reduces BUC risk; induces apoptosis and cycle blockade	Reduces risk in the population (especially men); inhibits T24 cell proliferation and induces apoptosis	1. Induces cell cycle G2/M block, inhibits PI3K/Akt pathway, increases Bax, decreases Bcl-2, triggers mitochondrial apoptosis [135].	In vitro
*Formononetin*	The main active ingredient of *Astragalus*, low to normal cytotoxicity	Synergizes with chemotherapy, inhibits metastasis	Inhibits proliferation and invasion; enhances gemcitabine efficacy; reverses EMT process	1. Selectively targets tumor cells and synergizes with gemcitabine [136].	In vitro and in vivo
				2. Reverses EMT by up-regulating E-cadherin, down-regulating N-cadherin, and inhibiting matrix metalloproteinase activity [137].	In vitro
*Quercetin*	Apple, tea, red wine source, low bioavailability	Induces apoptosis, sensitizes to radiotherapy	Inhibits cancer cell proliferation and induces apoptosis	1. Activation of the AMPK signaling pathway induces apoptosis in BUCs [138].	In vitro

**Table 7:** The Summary of the Effects of Alkaloids on BUCs, Including Their Sources, Clinical Applications, Effects on BUCs, and Mechanisms.

Alkaloid	Source and Nature	Efficacy	Author	Research Type	Effects on BUC	Mechanism
*Alperine*	The quinoline alkaloid active ingredients extracted from the leaves and stems of the traditional Chinese medicines *Sophora flavescens* A. and *Sophora flavescens* L. (Fabaceae).	Possesses broad anti-tumor, antibacterial, antipyretic, anti-injury, and anti-inflammatory pharmacological capabilities.	Qiu M et al. [155]	In vitro	At high doses, it can inhibit the activity of BUCs, while at low doses, it inhibits the migration, invasion, and adhesion of BUCs.	Inhibits the expression of MMP-2 and MMP-3 and promotes the expression of TIMP-4, thereby participating in the inhibition of ALO-induced BUCs migration, invasion, and adhesion.
*Berberine*	Isoquinoline alkaloids isolated from *Coptis chinensis*	1. Treatment of diarrhea and gastroenteritis 2. Inhibition of gastric cancer cells	Li Q et al. [143]	In vitro	Inhibited the proliferation, migration, invasion, and cell cycle progression of BUCs, and promoted their apoptosis.	Has a similar synergistic effect with HER2 inhibitors, leading to downregulation of HER2/PI3K/AKT protein expression.
			Gao X et al. [144]	In vivo	Antiproliferative effects promoting apoptosis and cell cycle arrest	Inactivating the PI3K/Akt pathway to downregulate Rad51 expression enhances the cytotoxicity, apoptosis, and migration inhibition of gemcitabine-induced BUCs, while also attenuating gemcitabine-induced Akt phosphorylation.
			Xia Y et al. [145]	In vivo and in vitro	Effectively inhibits the proliferation, migration, and invasion of BUCs and induces apoptosis or senescence of BUCs.	By upregulating miR-17-5p to inhibit Janus kinase 1 (JAK1)-STAT3 signaling, miR-17-5p can directly bind to the 3′UTR of JAK1 and STAT3, downregulating their expression and exerting an antitumor effect on BUCs.
			Han C et al. [146]	In vivo and in vitro	Inhibition of cell viability, colony formation, and proliferation	1. The synergistic mechanism that stimulates the expression of P21 and P27 proteins and downregulates the expression of Cyclin D, Cyclin A2, and CDK2 proteins induces cell cycle arrest in the S phase. 2. Inhibits epithelial–mesenchymal transition (EMT) and rearranges the cytoskeleton to inhibit cell metastasis. 3. Downregulating antioxidant genes such as Nrf2, HO-1, SOD2, and GPX-1 to increase intracellular reactive oxygen species (ROS) levels. Upon ROS accumulation, the Bax/Bcl-2 ratio increases, triggering the intrinsic apoptosis pathway. 4. The ROS accumulation mediated by this process negatively regulates the NF-κB pathway to exert an antitumor effect.
*Betalains*	Natural pigments present in beetroot *Beta vulgaris* var. *rubra* L. (BVr)	It has antihypertensive and hypoglycemic activity, as well as excellent antioxidant activity, and exhibits antiproliferative activity against cancer cell lines.	Scarpa ES et al. [156]	In vitro	Inhibition of T24 BUCs proliferation	Activate the extrinsic apoptosis pathway, enhance caspase 8 activity, and induce extrinsic cell apoptosis.
*Capsaicin*	An active ingredient in chili peppers (8-methyl-N-vanillyl-6-nonanamide)	Inhibits gastric cancer, hepatocellular carcinoma, etc.	Lin MH et al. [147]	In vitro	Inhibits the growth of BUCs, reduces proliferation, weakens migration, and prolongs the cell cycle process.	1. Enhances apoptosis to downregulate tNOX expression and reduce BUCs growth. 2. Reduces the expression levels of several proteins involved in the cell cycle process, leading to an increase in cell doubling time and cell cycle arrest. 3. Inhibits ERK activation, reduces the phosphorylation of p21 and FAK, thereby reducing cell migration. 4. Inhibits SIRT1 to suppress the growth of BUCs.
*Cernumidine*	Guanidine alkaloids isolated from water-ethanol extracts of *S. cernuum* leaves.		Miranda MA et al. [157]	In vitro	Combination therapy with cisplatin: CER has cytotoxic properties and can make BUCs more sensitive to cisplatin.	1. Inhibited cell migration in the T24 BUCs line. 2. Reduced MMP-2/9 and EGFR protein levels and inhibited ERK1/2 phosphorylation. 3. Increased the Bax/Bcl2 ratio and activated the apoptotic mitochondrial signaling pathway in the T24 BUCs line (Bax is a pro-apoptotic protein, while Bcl-2 is an anti-apoptotic protein).
*Ellipticine*	Natural alkaloids are isolated from the leaves of plants belonging to the family *Rubiaceae*.	Effective in treating stage II late-stage breast cancer	Tao S et al. [158]	In vitro	Induces cell death and G2/M cell cycle arrest, and reduces the migratory ability of T-24 BUCs in a dose- and time-dependent manner.	Activation of the ATM serine/threonine kinase pathway triggered by *Ellipticine*
*Fangchinoline*	A major dibenzylisoquinoline alkaloid found in the plant *Stephania tetrandra*.	1. Inhibits acetylcholinesterase and glutamate release in neurological diseases. 2. Antioxidant, showing protective effects against oxidative cell damage. 3. Inhibits the growth of various cancers, including lung cancer, breast cancer, and prostate cancer, and induces autophagy-mediated cell death in hepatocellular carcinoma cells. 4. Inhibits P-glycoprotein activity in multidrug-resistant human cancer cells to reverse multidrug resistance.	Fan B et al. [159]	In vitro	Has anti-tumor effects on BUC	It can inhibit proliferation and induce caspase-dependent apoptosis in BUC, and for the first time, reveals that Fcn induces protective autophagy in BUC, inhibits the mTOR pathway, and ultimately induces energy impairment in BUC.
*Harmine*	β-carboline alkaloids extracted from *Pergamum harmala* seeds	1. Specific inhibition of protein kinase DYRK1A kinase activity 2. Anti-tumor and anti-depressant 3. Prevention of type 1 and type 2 diabetes 4. Inhibition of cancer cells originating from the breast, lung, bone, and pancreas	Hai-Rong C et al. [160]	In vivo	Inhibits tumor growth and tumor angiogenesis, showing promising therapeutic potential for BUC treatment.	By inhibiting VEGF-mediated tumor angiogenesis, cell proliferation, migration, invasion, and tube formation through multispectral suppression, and by inducing apoptosis in BUCs through the activation of the caspase-dependent apoptosis pathway, it downregulates the downstream vascular endothelial growth factor receptor 2 (VEGFR2) kinase pathway, significantly inhibiting VEGFR2 kinase activity and thereby suppressing tumor development signals.
*Homohar-ringtonine*	A plant-derived alkaloid isolated from *Cephalosporium*		Wu Q et al. [161]	In vivo And in vitro	Effectively inhibits the proliferation, colony formation, cell adhesion, and migration of BUCs while inducing apoptosis and cell cycle arrest.	1. BUCs undergo apoptosis and arrest at the G2/M phase after HHT treatment. 2. HHT inactivates integrin α5/β1, downregulates FAK/Src, inhibits their phosphorylation, and suppresses cell adhesion, proliferation, and migration. 3. Inhibits the MAPK/Erk and PI3K/Akt signaling pathways, suppressing cell survival and proliferation.
*Liensinine*	Natural isoquinoline alkaloids isolated from the seed embryos of *Nelumbo nucifera* Gaertn	1. Anti-lipid abnormalities, antioxidant, and anti-inflammatory effects 2. Anti-gastric cancer, breast cancer, and prostate cancer	Jiang H et al. [149]	In vitro	Promoting BUCs senescence and inhibiting proliferation	1. Inhibiting the CDK2/4 and PI3K/AKT pathways in BUCs to promote senescence and inhibit proliferation 2. In the anti-BUCs activity, cyclin-dependent kinase 2 (CDK2) and CDK4 proteins are the core targets of LIEN, with CDK2 exhibiting more stable binding to LIEN than CDK4 3. Reducing the expression of phosphorylated AKT1 protein and degrading AKT1 itself to inhibit tumor growth, thereby regulating the activity of the PI3K/AKT signaling pathway; LIEN may also exert its anti-BUCs activity by blocking the PI3K/AKT signaling pathway and reducing p-AKT levels to activate the FOXO3a signaling pathway.
*Lycorine*	Pyrroloquinoline alkaloids extracted from the genus *Amaryllis*	1. Antiviral and antimalarial properties. 2. Alters the organization of the actin cytoskeleton in cancer cells, thereby significantly impairing cancer cell proliferation and migration and inhibiting the growth of various tumors. 3. Treats leukemia and prostate cancer.	Wang C et al. [162]	In vivo	Has a strong cytotoxic effect on T24 cells.	The PI3K/Akt pathway is involved in mediating the inhibitory effects of *Lycorine* on cell proliferation and survival by upregulating its negative regulator, PTEN, thereby reducing the expression of key factors such as phosphorylated Akt, and ultimately increasing the expression level of cleaved caspase-3.
*Matrine*	One of the key tetracyclic quinoline alkaloids isolated from the roots of *Sophora flavescens*	Reduces liver fibrosis, anti-inflammatory, anti-allergic, anti-viral, cardiovascular protection, and anti-cancer potential	Li L et al. [150]	In vivo	It has no adverse effect on the growth of normal urinary tract epithelial cell lines, but it can inhibit the growth, invasion, and migration of BUCs.	By upregulating the expression of LINC00472/PDCD4, it can block the PTEN/PI3K/AKT pathway in BUCs.
			Wang L et al. [151]	In vivo and in vitro	It is possible to use it as a therapeutic intervention for BUC caused by exposure to arsenic trioxide.	Reduction in NADPH oxidase 2 (NOX2) levels and inhibition of bladder epithelial cell transformation
			Su Y et al. [152]	In vitro	Selectively inhibits the growth of BUCs without inhibiting the growth of healthy bladder tissue.	1. Inhibits the proliferation, apoptosis, and cell cycle arrest of BUCs by activating the PI3K/AKT/FoxO3a signaling pathway through abnormal activation. 2. Exerts a growth inhibitory effect on BUCs by promoting the production of intracellular ROS and activating the mitochondrial ROS-mediated signaling pathway.
*Piperlongu-mine*	The main bioactive alkaloid in long peppers, with a well-characterized structure (C17H19NO5)	Highly selective cytotoxic compounds are effective against cancer cells, including hepatocellular carcinoma, breast cancer, gastric cancer, and non-small cell lung cancer.	Liu D et al. [163]	In vivo and in vitro	Effectively inhibits the development of BUCs in vitro and in vivo	1. Prioritize the inhibition of BUCs invasion through the ROS, Erk, and PKC signaling pathways, as well as the EMT/F-actin pathway. 2. Reverse the epithelial–mesenchymal transition (EMT) process by downregulating the E-cadherin transcription repressor in T24 xenografts. 3. PL also affects the cytoskeleton: plate-like pseudopod formation is significantly reduced in BUCs, and F-actin intensity is decreased.
*Securinine*	A natural alkaloid isolated for the first time from *Securinega suffruticosa*	Central nervous system stimulant, anti-inflammatory, antimalarial, anti-toxoplasmic, antibacterial, and antifungal activity, etc.	Xie L et al. [164]	In vivo and in vitro	Inhibits the proliferation, migration, and invasion of BUCs, induces apoptosis of BUCs in vitro, and delays the growth of xenograft tumors of BUCs in vivo.	Exerting anti-BUCs effects by activating the p38 and JNK signaling pathways and inhibiting the Wnt/β-catenin signaling pathway.
*Tetrandrine*	A double-benzyl isoquinoline alkaloid isolated from the traditional Chinese medicine *Stephaniae*	1. Anti-hypertension, anti-silicosis, and anti-arrhythmia 2. Anti-prostate cancer, colon cancer, stomach cancer, etc.	Zhang Y et al. [165]	In vitro	Inhibiting the migration and invasion of BUC 5637 and T24 cells	Reversing EMT by increasing the expression of E-cadherin and decreasing the expression of N-cadherin, vimentin, and Slug in a dose-dependent manner, as well as by downregulating the expression of Gli-1, can also reverse EMT in BUCs.
*Theophylline*			Chen Z et al. [166]	In vitro	Inhibits BUC proliferation and promotes BUC apoptosis.	A *Theophylline*-controlled RNAi-based genetic switch was created that can silence TINCR in a dose-dependent manner. Both the RNAi-OFF and ON switches can be used to quantitatively control the expression of TINCR in BUC to inhibit the progression of BUC.
*Trigonelline*			Kao CC et al. [153]	In vivo and in vitro	Candidate drugs for the treatment of drug-resistant BUC	1. Inhibits Nrf2-mediated anti-apoptotic pathways [167] 2. Inhibits PI3K, Akt, and PLCγ signaling pathways to exert anticancer functions 3. Forms a stable complex with TGFβ3, participates in the downregulation of TGY characteristics, significantly reduces bladder tumorigenicity, particularly tumor spheroid formation capacity, cisplatin resistance, and CAF conversion capacity 4. Reduces TGFβ3, IL-6, and VEGF production in CAF-containing tumor-like cells
*Triacanthine*	Alkaloids obtained from locust trees (*Gleditsia* spp.)	1. Treatment of boils, scabies, skin inflammation, stroke, cough, and asthma 2. Reduced proliferation of breast cancer, lung cancer, and esophageal cancer cell lines	Shin SS et al. [154]	In vivo and in vitro	Inhibition of BUC proliferation, migration, and invasion in vitro and in xenograft tumors	1. Effectively inhibits growth and G1-S cell cycle transition both in vivo and in vitro, and induces apoptosis and autophagy in BUCs: *Triacanthine* inhibits G1-S transition by blocking the formation of complexes between cyclin-independent kinases and cyclin proteins, thereby upregulating the cell cycle inhibitors p21WAF1 and p27KIP1. 2. Induces caspase-dependent apoptosis and autophagy through an exogenous pathway: Upregulates early response kinases, extracellular signal-regulated kinases (ERK), and Janus kinases (JNK). 3. Effectively inhibits MMP-9 by blocking transcription factor binding: Inhibits the binding of AP-1, Sp-1, and NF-κB to MMP-9.

**Table 8:** The summary of functions, effects on BUCa, and the relating mechanism of Another Natural Product.

Natural Product	Source and nature	Efficacy	Research Type	Effects on BUC	Mechanism of Action
*Rhodopsin*	constituent present in various plants such as *Aloe vera* and *Polygonum multiflorum*	anti-inflammatory, antibacterial, and antitumor properties	In vitro	exhibits concentration-dependent inhibitory effects on the proliferation and invasion of T24 and 5637 BUCs	Inhibits Notch1 pathway(mRNA and protein levels of Jagged1, VEGF, VEGFR2 and MMP2 are significantly inhibited) [169]
*Arbutin*	distributed in the plant, such as *Asteraceae* and *Rhododendronaceae*	a natural antioxidant, inhibits the onset of ferroptosis and ameliorates high-fat diet-induced NAFLD in vivo and in vitro	In vitro	reduces the proliferation of TCCSUP line in a concentration and time-dependent manner	Inactivates ERK and upregulates p21 [171]
*Tanshinone IIA*	extracted from the roots of the traditional Chinese medicine *Salvia miltiorrhiza*	a protective effect against atherosclerosis Processes biological activities such as anti-inflammatory, antioxidant, and neuron-protective	In vitro	inhibits the migration and invasion of BUCa cells	inhibits the STAT3 pathway; down-regulating CCL2 expression in BUCa cells; inhibits of EMT [172]
*Garcinia acid*	derived from the natural resin *Garcinia cambogia*	antiproliferative and pro-apoptotic effects on certain tumor (e.g., breast cancer, prostate cancer, etc.) cell lines	In vitro	induces apoptosis in BUCs lines T24 and UMUC3 in a ROS-dependent manner	activates the JNK pathway; generates ROS, triggers the intrinsic apoptotic pathway; inhibits NF-κB, achieved through the inhibition of IκB-α [177]

## Data Availability

No new data were created or analyzed in this study. Data sharing is not applicable to this article.

## Abbreviations

The following abbreviations are used in this manuscript:

BUC	Bladder Urothelial carcinoma
BUCs	Bladder Urothelial carcinoma cells
MIBC	Muscle-invasive bladder carcinoma
TURBT	Transurethral resection of bladder tumor
NMIBC	Non-muscle-invasive bladder carcinoma
ROS	Reactive oxygen species
ΔΨm	Mitochondrial membrane potential
Mta-1	Metastasis-associated gene 1
ART	*Artesunate*
ATR	Atractylenolide
CDK1	Cyclin-dependent kinase 1
MMP-9	Matrix metalloproteinase-9
ER	Endoplasmic reticulum
TCC	Transitional cell carcinoma
JB	*Jolkinolide B*
TrxR1	Thioredoxin Reductase 1
mTOR	Mammalian target of rapamycin
mTORi	mTOR inhibitors
ERK	Extracellular-regulated kinase
GPX4	Glutathione peroxidase 4
GPX1	Glutathione peroxidase 1
AA	*Abietic acid*
HO-1	Heme oxygenase-1
NTUB1	Human bladder cancer cell line
BA	*Betulinic acid*
SLC7A11	Solute carrier family 7 member 11
EMT	Epithelial–mesenchymal transition
TRAIL	TNF-related apoptosis-inducing ligand
PD	*Platycodin D*
Trop-2	Trophoblast surface antigen 2
HER-2	Human epidermal growth factor receptor 2
IGF-2	Insulin-like growth factor 2
IGF-1R	Insulin-like growth factor-1 receptor
MMPs	Matrix metalloproteinases
NF-κB	Nuclear factor kappa-B
CHOP	C/EBP homologous protein
Cyt-c	cytochrome C
EGCG	Eigallocatechin gallate
ISL	Isoliquiritigenin
LCA	licochalcone A
ECN	echinatin
JAK1	Janus Kinase
Nrf2	Nuclear factor E2-related factor 2
FAK	Focal adhesion kinase
TRPV1	Transient receptor potential vanilloid 1
LIEN	*Liensinine*
MAT	*Matrine*
TGN	*Trigonelline*
TGY	TGFβ3/GLI2/YAP1
TGFβ3	transforming growth factor-β3
VEGF	Vascular endothelial growth factor
JNK	Janus kinases
AP-1	Activating protein-1
STAT3	Signal transducer and activator of transcription 3
AC	*Asiaticoside*
BCG	Bacillus Calmette-Guérin
VEGFR2	Vascular endothelial growth factor receptor 2
Tan-IIA	Tanshinone IIA
GA	Garcinia acid

## Appendix A

**Table A1:** Summary of clinical trials on the combination of *paclitaxel* and other chemotherapy drugs.

Interventions	Cancer Types	Study Date	Study Types	Phase	PMID	NCTnumber
DRUG: Tislelizumab|DRUG: Nab *paclitaxel*	Muscle-invasive bladder cancer	11 July 2020	Interventional	Phase II	PMID: 39777450 PMID: 38762368	NCT04730219
DRUG: Trastuzumab biosimilars (Herzuma), Gedatolisib	HER2-positive Breast Cancer| Metastatic Breast Cancer	3 December 2019	Interventional	Phase II	PMID: 37385153	NCT03698383
DRUG: *paclitaxel*|RADIATION: Radiation Therapy|BIOLOGICAL: Trastuzumab	Bladder Urothelial Carcinoma|Stage I Bladder Cancer AJCC v6 and v7|Stage II Bladder Cancer AJCC v6 and v7|Stage III Bladder Cancer AJCC v6 and v7	26 July 2005	Interventional	Phase II	PMID: 37442672	NCT00238420
DRUG: Nivolumab + Nab-*paclitaxel*|DRUG: Nivolumab	Muscle-Invasive Bladder Carcinoma	27 January 2022	Interventional	Phase I/II	PMID: 39241203	NCT04876313
DRUG: Gemcitabine|DRUG: Docetaxel	Urothelial Carcinoma Bladder|Bladder Cancer	29 July 2020	Interventional	Phase II	PMID: 38653234	NCT04386746
DRUG: Everolimus|DRUG: Everolimus|DRUG: *paclitaxel*	Transitional Cell Carcinoma|Bladder Carcinoma|Urothelial Carcinoma	1 December 2010	Interventional	Phase II	PMID: 35438782	NCT01215136
DRUG: anti-PD-L1 antibody|DRUG: albumin bound *paclitaxel*	Advanced Urothelial Carcinoma	10 September 2020	Interventional	Phase I	PMID: 39418340	NCT04603846
